# IK is essentially involved in ciliogenesis as an upstream regulator of oral-facial-digital syndrome ciliopathy gene,* ofd1*

**DOI:** 10.1186/s13578-023-01146-9

**Published:** 2023-10-28

**Authors:** Hye In Ka, Mina Cho, Seung-Hae Kwon, Se Hwan Mun, Sora Han, Min Jung Kim, Young Yang

**Affiliations:** 1 Research Institute of Women’s Health, Sookmyung Women’s University, Seoul, 04312 South Korea; 2 Chronic and Metabolic Diseases Research Center, Sookmyung Women’s University, Seoul, 04312 South Korea; 3 Seoul Center, Korea Basic Science Institute, Seoul, 02841 South Korea; 4 Department of Biological Sciences, Sookmyung Women’s University, Seoul, 04312 South Korea

**Keywords:** Ciliopathy, Ciliogenesis, IK, Oral-facial-digital syndrome, OFD1

## Abstract

**Background:**

The cilia are microtubule-based organelles that protrude from the cell surface. Abnormalities in cilia result in various ciliopathies, including polycystic kidney disease (PKD), Bardet-Biedl syndrome (BBS), and oral-facial-digital syndrome type I (OFD1), which show genetic defects associated with cilia formation. Although an increasing number of human diseases is attributed to ciliary defects, the functions or regulatory mechanisms of several ciliopathy genes remain unclear. Because multi ciliated cells (MCCs) are especially deep in vivo, studying ciliogenesis is challenging. Here, we demonstrate that *ik* is essential for ciliogenesis in vivo.

**Results:**

In the absence of *ik*, zebrafish embryos showed various ciliopathy phenotypes, such as body curvature, abnormal otoliths, and cyst formation in the kidney. RNA sequencing analysis revealed that *ik* positively regulated *ofd1* expression required for cilium assembly. In fact, depletion of *ik* resulted in the downregulation of *ofd1* expression with ciliary defects, and these ciliary defects in *ik* mutants were rescued by restoring *ofd1* expression. Interestingly, *ik* affected ciliogenesis particularly in the proximal tubule but not in the distal tubule in the kidney.

**Conclusions:**

This study demonstrates the role of *ik* in ciliogenesis in vivo for the first time*.* Loss of *ik* in zebrafish embryos displays various ciliopathy phenotypes with abnormal ciliary morphology in ciliary tissues. Our findings on the *ik*–*ofd1* axis provide new insights into the biological function of *ik* in clinical ciliopathy studies in humans.

**Supplementary Information:**

The online version contains supplementary material available at 10.1186/s13578-023-01146-9.

## Background

Primary cilia are microtubule-based organelles that protrude from eukaryotic cell surfaces. In most cell types, primary cilia are highly dependent on the phase of cell cycle because they are dynamically regulated during cell cycle progression. They begin to form in the G0 and G1 phases, disassemble typically from the S/G2 phase, and finally undergo rapid resorption before entry into mitosis [1]. Primary cilia function as cellular antennae and intermediate external stimuli to spatiotemporally determine cell fate in the homeostasis of tissue development [2, 3]. Thus, dysfunction of primary cilia in tissues or organs leads to a variety of genetic pleiotropy and syndromic disorders, commonly termed ciliopathies, such as polycystic kidney disease, Bardet–Biedl syndrome, nephronophthisis, and retinitis pigmentosa [4–8]. Ciliopathies are induced by various factors, including genetic mutations, loss of intraflagellar transport proteins, improper docking of basal body, and abnormal pre-mRNA splicing of ciliary component genes [9–14]. Although an increasing number of human diseases are attributed to ciliopathies, causative factors and their mechanistical and molecular functions in ciliary defects are largely unexplored in vivo. Therefore, elucidating the molecular correlations and functions of various genes associated with ciliopathy are challenging.

The nuclear protein IK, also called as RED because of existence of sequence repeated arginine(R), glutamic acid(E) and aspartic acid(D) residues, is necessary for cell progression in mitosis and attributes in genomic stability by contributing to pre-mRNA splicing. Structurally, IK binds with SMU1 and the interacted two proteins perform the coordinated functions as a dimer complex with stabilizing reciprocally. Thus, lack of one protein makes to degrade reciprocal protein and leads to perturbed cellular function [15].In mitosis, IK recruits a core mitotic checkpoint protein mitotic arrest deficient 1 (MAD1) at the spindle poles and functions in mitotic progression of the spindle assembly checkpoint [16, 17]. Furthermore, IK-depleted cells show suppressed activity of PP1/PP2A which subsequently contributes to the sustained activity of Aurora kinase A, a member of mitotic serine/threonine kinase [18]. Meanwhile, IK also known to be required for activation of spliceosomal B complexes as it functions in the pre-mRNA splicing of short introns [19]. Although the expression level of *ik* is different in various tissues, the precise role of *ik* in specific tissues remains still unclear. Homozygous knockout of *ik* in mice results in embryonic lethality and we previously demonstrated that depletion of *ik* impairs the development of skeletal muscle in vivo using zebrafish model [20]. However, further studies on the physiological role of IK in vivo need be more required.

Oral-facial-digital 1 (*ofd1*) is the first gene reported in oral-facial-digital syndrome (OMIM 311200) [21–24] and encodes OFD1 protein located in the centrosome and basal body of primary cilia [21]. During ciliogenesis, it modulates the length of centriole and formation of distal appendages in the mother centriole [25–27]. Thus, the absence of o*fd1* induces the abnormal elongation of distal regions of centrioles, and the long centrioles form structurally destabilized microtubules with abnormal post-translational modifications [27]. In human, mutations in o*fd1* result in abnormal ciliary morphology and occurs OFD syndrome characterized by malformations of the face, oral cavity, and digits [28–30]. The patients with OFD syndrome present with several features common to ciliopathies, such as cystic disease and patterning abnormalities [28]. To date, the studies of ciliary dysfunction in the absence of OFD1 has been described using diverse in vitro and in vivo models [25, 27, 31–34]. In zebrafish, loss of *ofd1* results in bent body axes, edema, and perturbed intravascular fluid flow in Kupffer’s vesicles with shorted and disrupted axonemes in cilia [31]. However, the molecular mechanisms controlling the expression of *ofd1* beyond cilium assembly are poorly understood.

Here, we found that IK is positively associated with *ofd1* expression, which is required for cilium assembly. Depletion of IK downregulates *ofd1* expression and restoration of *ofd1* expression overcomes IK-depletion-induced ciliary defects. Our findings show that IK–OFD1 axis is critical for normal ciliary development in vivo and provide new biological insights into clinical human ciliopathy.

## Results

### Zebrafish *ik* mutants display ciliopathy-like phenotypes

**Fig. 1:**
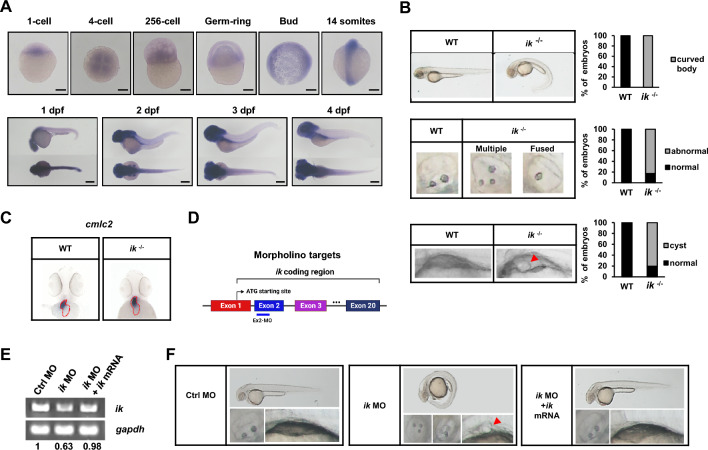
zebrafish *ik* mutants display ciliopathy-like phenotypes (**A**) Whole-mount in situ hybridization (WISH) analysis of zebrafish *ik* mRNA at different developmental stages (1-cell, 4-cell, 256-cell, Germ-ring, Bud, 14 somites, 1 dpf, 2 dpf, 3 dpf, and 4 dpf). Scale bar, 250 μm (**B**) Brightfield microscopic images of the body curvature, otolith, and pronephric cysts pronephros in WT embryos and *ik* mutants (*ik* ^−/−^) at 2 dpf. Pronephric cyst of *ik* mutants is marked with red arrowhead. Stacked bar graph displays the percentage of embryos with a ventrally curved body, abnormal otolith phenotype, and cyst formation (**C**) WISH analysis of *cmlc2* for the location of whole heart in WT embryos and *ik* mutants at 2 dpf (**D**) Schematic representation of the coding region of zebrafish *ik*. The target region (exon 2) of the morpholino employed in this study was represented using a blue line (**E**) RT-PCR analysis of *ik* mRNA expression in control morphants (MO), *ik* MO, and *ik* MO/*ik* mRNA co-injected embryos at 2 dpf. *gapdh* served as a normalization control and band intensity ratio of *ik*/*gapdh* mRNA expression was marked (**F**) Brightfield microscopic images of control MO, *ik* MO, and *ik* MO/*ik* mRNA co-injected embryos at 2 dpf. Whole body, otolith, and kidney cyst were captured. Pronephric cysts of *ik* MO are marked with red arrowhead

Zebrafish is considered to be the most suitable in vivo model to investigate the function of *ik* because homozygous knockout of *ik* is embryonic lethal in mice but not in zebrafish embryos. To explore the function of IK in vivo, we examined the spatiotemporal expression of *ik* during embryogenesis in zebrafish using whole-mount in situ hybridization (WISH) and quantitative RT-PCR (Fig. 1A, Additional file 1: Fig. S1). *ik w*as expressed from 1-cell close to the blastoderm margin, but the overall maternally expressed level was significantly low (approximately 14 somites). However, after the period of maternal to zygotic transition (MZT) (approximately 21 h after fertilization in zebrafish), *ik* expression became intense (after 1 day post fertilization), specifically in organs from the mesodermal lineage, including the head, pronephros, and muscle. During the zygotic stage*, ik* was ubiquitously distributed in the tailbud and became progressively enriched, particularly in ciliated tissues such as the head and pronephric duct (Additional file 2: Fig. S2). These spatiotemporal expression analyses show that *ik* expression is much higher at the zygotic stage than at the early stage and imply that IK may have a functional role in ciliated tissues of zebrafish. Next, to characterize the role of IK in vivo, we analyzed the phenotypes of previously generated homozygous *ik* knockout zebrafish mutants using CRISPR/Cas9 system [20] and observed that *ik* mutants exhibited ciliopathy phenotypes. At 2 days post fertilization (dpf), *ik* mutants showed a distinct body curvature of 100% (n = 112) and abnormal otolith phenotypes such as fused or multiple form (83%; n = 93 out of 112) (Fig. 1B). Also, cyst formation in pronephros was observed in 80% (n = 90 out of 112). In addition to these phenotypes, the randomized left–right (LR) axis of the heart looping is known as a major ciliopathy phenotype caused by defects in motile cilia in Kupffer’s vesicles during early development of zebrafish [35] because ciliated organ Kupffer’s vesicles determine the LR symmetry of the brain, gut, and heart at the early stage. To determine the alteration in the LR asymmetry patterning in *ik* mutants, we performed WISH analysis to detect LR asymmetry in the heart using cardiac myosin light chain 2 (*cmlc2)* as a marker for the whole heart of zebrafish embryos. Wild-type (WT) embryos and *ik* mutants showed no differences in LR organization, with a correct S-shaped loop (D-loop) (Fig. 1C). These results indicate that loss of *ik* causes major ciliopathy phenotypes but is not involved in determining LR asymmetry. Next, we depleted *ik* expression in zebrafish embryos using morpholino antisense oligonucleotides (MOs) targeting exon 2 at the one-cell stage to perform the rescue experiment (Fig. 1D, E) [20, 36]. *ik* morphants revealed various pleiotropic ciliopathy phenotypes, including body axis curvature (74.6%; n = 50 out of 67), otolith defects (50%; n = 11 out of 22), and pronephric cysts (85.71%; n = 30 out of 35) (Fig. 1F). To verify the specificity of *ik* MO knockdown, we additionally co-injected synthetic mRNA encoding full-length wild-type *ik* with MO. The co-injected *ik* mRNA rescued all defective ciliary phenotypes of *ik* morphants revealing the possibility of off-target effects was excluded.

### Loss of *ik* results in abnormal ciliary morphology

**Fig. 2:**
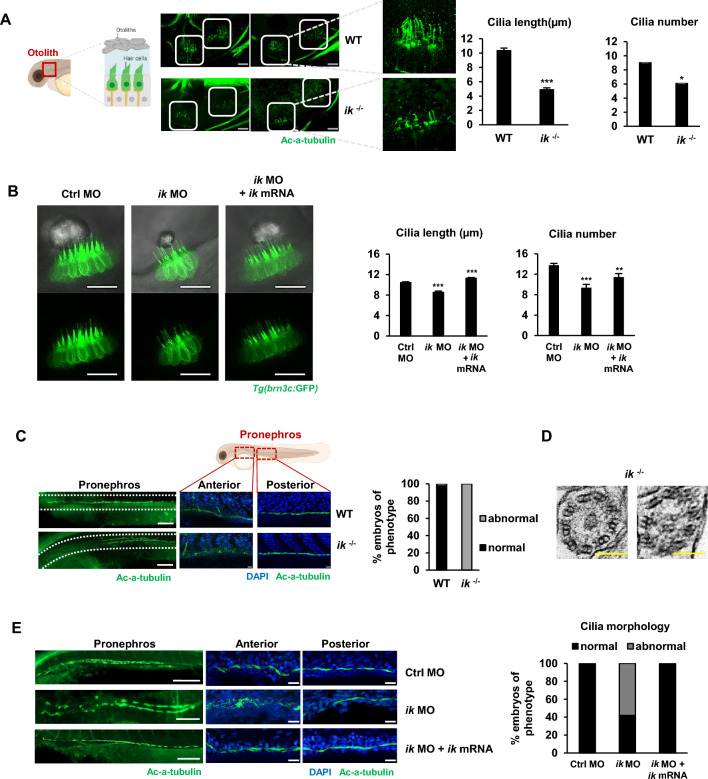
Loss of *ik* results in abnormal ciliary morphology (**A**) Whole-mount immunostaining of cilia in the inner ear (white boxes) using anti-acetylated-α-tubulin (green) of WT embryos (n = 3) and *ik* mutants (n = 5) at 2 dpf. Scale bar, 20 μm. Quantified graphs of cilia lengths and numbers in the inner ear between WT embryos and *ik* mutants at 2 dpf. *p < 0.05, ***p < 0.001 (**B**) Confocal microscopic images of control MO, *ik* MO, and *ik* MO/*ik* mRNA co-injected *Tg(brn3c:GFP)* embryos at 3 dpf. Scale bar, 20 µm. Quantification of ciliary length and number in the inner ear of control MO (n = 12), *ik* MO (n = 17), and *ik* MO/*ik* mRNA co-injected embryos (n = 10). **p < 0.01, ***p < 0.001 (**C**) Whole-mount immunofluorescence (left) of overall cilia in the pronephros in WT and in *ik* mutants at 2 dpf using anti-acetylated-α-tubulin (green). Scale bar, 100 µm. Enlarged view (right) of pronephric cilia in proximal and distal tubules stained with anti-acetylated-α-tubulin (green) and nuclei stained with DAPI (blue) at 2 dpf WT embryos and *ik* mutants. Scale bar, 20 µm. Stacked bar graph displays the percentage of embryos of cilia phenotype in the anterior pronephric duct of WT embryos and *ik* mutants at 2 dpf. (**D**) TEM results showing the ultrastructure of cilia in the pronephric duct of *ik* mutants. Cross-section showing the “9 + 2” configuration (black circles). Scale bar, 100 nm (**E**) Whole-mount immunofluorescence (left) of overall cilia in the pronephros of control MO, *ik* MO, and *ik* MO/*ik* mRNA co-injected embryos at 3 dpf with anti-acetylated-α-tubulin (green). Scale bar, 100 µm. Enlarged view (right) of pronephric cilia in the anterior and posterior pronephric ducts stained using anti-acetylated-α-tubulin (green) and nuclei stained with DAPI (blue) at 2 dpf control MO, *ik* MO, and *ik* MO/ *ik* mRNA co-injected embryos. Scale bar, 20 µm. Stacked bar graph displays the percentage of embryos based on ciliary morphology in the anterior pronephric duct (percentage of abnormal ciliary morphology of embryos: control MO, 0%; *ik* MO, 58%; *ik* MO + *ik* mRNA, 0%)

To determine the mechanism underlying ciliopathy phenotypes in *ik*-depleted embryos, we next focused on studying the impact of *ik* on ciliogenesis and abnormal ciliogenesis in hair cells was confirmed in *ik* mutants by immunostaining for *ac-α-tubulin,* a specific marker of cilia. In hair cells of *ik* mutants, the number of cilia was reduced by approximately 50% of WT embryos, and their length was shortened by approximately 30% (Fig. 2A). Furthermore, we used *Tg(brn3c:GFP)* zebrafish line with ciliary bundles on hair cells labeled with green fluorescent protein (GFP). Because the *brn3c* gene, a member of the POU (Pit-Oct-Unc) domain transcription factor *brn3* subfamilies (*brn3a, brn3b, and brn3c*), is selectively expressed in auditory and vestibular hair cells, the *Tg(brn3c:GFP)* line serves as an appropriate model for studying cilia in hair cells. We observed *ik* morphant *Tg(brn3c:GFP)* embryos showed a decrease in ciliary length and bundle number in hair cells (Fig. 2B). However, when full-mRNA of *ik* was co-injected into *ik* morphant *Tg(brn3c:GFP)* embryos, the abnormal ciliary phenotypes of *ik* morphant *Tg(brn3c:GFP)* embryos were almost completely rescued compared to those of the control group. Next, the effects of *ik* depletion on ciliogenesis were examined in the pronephros. *ik* mutants showed randomly misdirected cilia in pronephros, and we interestingly observed that disturbed cilia of the pronephros in *ik* mutants were specifically in the anterior but not in the posterior region (Fig. 2C). To determine whether aberrant ciliogenesis is owing to the alteration of the ultrastructure of motile cilia, we analyzed the organization or composition of axonemes of motile cilia using a transmission electron microscope (TEM). Similar to that of the WT embryos, *ik* mutants also had 9 + 2 arranged cilia in the pronephros (Fig. 2D, Additional file 3: Fig. S3). The result indicates that the abnormal ciliary morphology owing to the depletion of *ik* is not because of the damage to axoneme arrangement. Similar to that in *ik* mutants, 58% *ik* morphants showed randomly misdirected cilia in pronephros, and this abnormality was rescued by co-injection of *ik* mRNA (Fig. 2E). *ik* morphants also exhibited aberrant cilia only in the anterior region and not in the posterior region similar to *ik* mutants. These results show that IK functions specifically in the anterior region and not in the posterior region in the pronephros.

### *ik* mutants show impaired kidney development with abnormal ciliary morphology

**Fig. 3:**
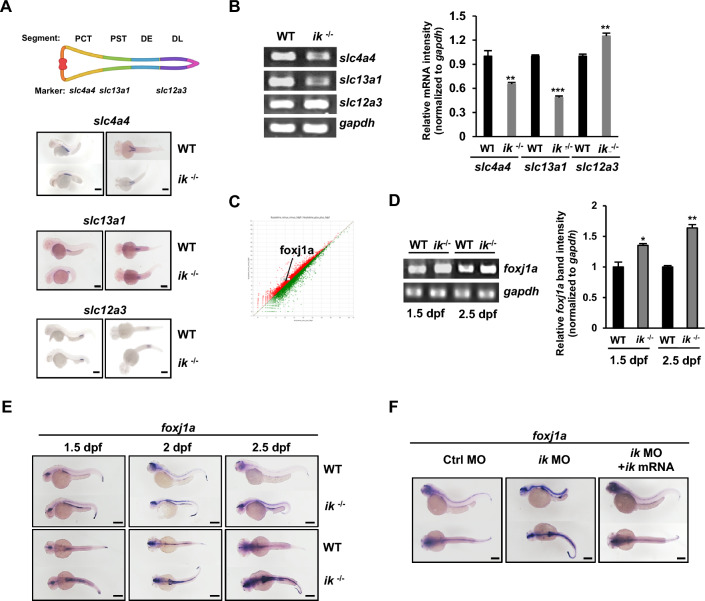
*ik* mutants show impaired kidney development with abnormal cilia morphology (**A**) WISH analysis of pronephric duct markers *slc4a4*, *slc13a1*, and *slc12a3* in WT embryos and *ik* mutants at 2 dpf. Scale bar, 250 μm (**B**) RT-PCR analysis of *slc4a4*, *slc13a1*, and *slc12a3* expression in WT embryos and *ik* mutants at 2 dpf. *gapdh* served as a normalization control. Relative bands intensities of *slc4a4*, *slc13a1*, and *slc12a3* normalized to *gapdh* intensity was graphically represented (in triplicates) using Image J software. **p < 0.01, ***p < 0.001 (**C**) Linear filter model showing correlation of overall gene expression based on RNA-seq data between WT and *ik* mutants at 3 dpf. The point of *foxj1a* plot is indicated. (**D**) RT-PCR analysis of *foxj1a* expression in WT embryos and *ik* mutants at 1.5 and 2.5 dpf. *gapdh* served as a normalization control. Relative band intensity of *foxj1a* normalized with respect to *gapdh* intensity is graphically represented (in triplicates) *p < 0.05, **p < 0.01 (**E**) WISH analysis of *foxj1a* in WT and *ik* mutants at 1.5, 2, and 2.5 dpf. Scale bar, 250 μm (**F**) WISH analysis of *foxj1a* in control MO, *ik* MO, and *ik* MO/*ik* mRNA co-injected embryos at 2.5 dpf. Scale bar, 250 μm

Tissues with ciliary defects generally show impaired development [37, 38] and abnormal primary cilia cause impaired kidney development, such as autosomal recessive polycystic kidney disease (ARPKD) [39]. Because *ik* mutants also revealed abnormal ciliogenesis in the pronephros, they may be defective in kidney development. Thus, we measured the spatial expression of the segment marker genes of the proximal tubule to examine whether the development of proximal tubule is abnormal. The expression of *slc4a4* in the proximal convoluted tubule (PCT) and *slc13a1* in the proximal straight tubule (PST) was reduced in *ik* mutants (Fig. 3A). Interestingly, the spatial mRNA expression of *slc4a4* in *ik* mutants showed shorter Y-shaped PCT structure than that of the WT. And spatial mRNA expression of the PST marker *slc13a1* also showed a shorter PST structure with weaker expression than that of the WT. However, the expression of distal late segment marker *slc12a3* was slightly increased in *ik* mutants, and the spatial expression of *slc12a3* showed an unaltered structure compared with that of the WT. The levels of segmental marker genes were confirmed by reverse transcription polymerase chain reaction (RT-PCR) (Fig. 3B). This result implies that *ik* mutants have abnormal kidney development, showing impaired ciliary morphology specific to the anterior but not the posterior region. *foxj1a*, essential gene for motile cilia formation in the kidney, is rapidly induced in response to impaired kidney development or cyst formation [40]. Therefore, we examined whether *foxj1a* expression was increased in *ik* mutants with abnormal kidney development and cyst formation. RNA sequencing (RNA-seq) analysis revealed that *foxj1a* expression was increased in *ik* mutants by 1.8 at log2-fold changes relative to that of the WT embryos (Fig. 3C), and this enhanced expression was also confirmed by RT–PCR (Fig. 3D). Additionally, the spatiotemporal expression of *foxj1a* was analyzed by WISH at 1.5–2.5 dpf (Fig. 3E). At 1.5 dpf, *foxj1a* expression was upregulated in ciliary tissues, including the pronephros, in *ik* mutants compared to that in WT embryos, and it was continuously maintained at 2.5 dpf. Next, we performed rescue experiments using co-injection of *ik* mRNA into *ik* morphants to examine whether overexpression of *ik* directly affected *foxj1* expression. Unlike *ik* morphants showing significantly enhanced *foxj1a* expression, *ik* morphants co-injected with *ik* mRNA showed *foxj1a* expression similar to that of the WT embryos (Fig. 3F).

### *ofd1*, a necessary gene for cilia assembly, is downregulated by *ik* depletion

**Fig. 4:**
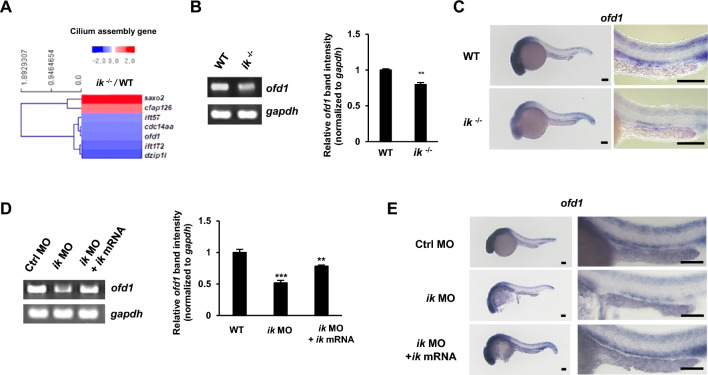
*ofd1*, a necessary gene for cilia assembly, is downregulated by the loss of *ik* (**A**) Heat map of seven differentially expressed cilium assembly genes at least 1.5 > log2 fold changes in *ik* mutants relative to those of WT embryos. Upregulated DEGs relative to the mean are indicated by red color. Downregulated DEGs are shown by blue color (scale bar, log 2 of mRNA ratio) (**B**) RT-PCR analysis of *ofd1* mRNA expression in WT embryos and *ik* mutants. *gapdh* served as a normalization control. Relative band intensity of *ofd1* normalized with respect to *gapdh* intensity is graphically represented (in triplicate) using Image J software. **p < 0.01 (**C**) WISH for *ofd1* in WT embryos and *ik* mutants at 1 dpf. Scale bar, 100 μm. Enlarged view of the pronephros in WT and *ik* mutants at 1 dpf. Scale bar, 150 μm (**D**) RT-PCR analysis of *ofd1* mRNA expression in control MO, *ik* MO, and *ik* MO/*ik* mRNA co-injected embryos at 1.5 dpf. *gapdh* served as a normalization control. Relative band intensity of *ofd1* normalized with respect to *gapdh* intensity is graphically represented (in three individual experiments) using Image J software. **p < 0.01, ***p < 0.001 (**E**) WISH for *ofd1* in control MO, *ik* MO, and *ik* MO/*ik* mRNA co-injected embryos at 1 dpf. Enlarged view of the pronephros in control MO, *ik* MO, and *ik* MO/*ik* mRNA co-injected embryos at 1 dpf. Scale bar, 100 μm

As *ik* was found to be essential for proper ciliogenesis with the development of ciliary tissues, RNA-seq data were analyzed to identify cilia assembly-related genes that were differentially expressed between WT and *ik* mutants. The log2-transformed transcriptome expression in RNA-seq of *ik* mutants was analyzed according to the strand-specific index with statistical significance set at *p* < 0.05. Differentially expressed genes involved in cilium assembly (Gene Ontology(GO):0060271) were also identified (Fig. 4A). Among the filtered cilium assembly genes, including *ift57, ift172, dzip1l* and *ofd1*, we focused on *ofd1* gene with 0.59 at log2-fold changes as a target of *ik*, because the previously known physiological phenotypes of *ofd1* mutants were considerably similar to *ik* mutants showing ciliopathy such as body curve, otolith abnormalities and a wide spectrum of malformations [31]. Indeed, *ofd1* expression was downregulated in *ik* mutants (Fig. 4B) and was spatially reduced in the head and pronephros (Fig. 4C). To exclude the possibility of off-target effects, rescue experiments were additionally performed. The downregulated *ofd1* expression in *ik* morphants was enforced by co-injection of *ik* mRNA as indicated by RT-PCR (Fig. 4D) and WISH experiments (Fig. 4E). This means that *ofd1* is a downstream target of *ik *in vivo. Next, we performed the rescue experiment in vitro to confirm that *IK* functions as a transcriptional upstream regulator of *OFD1*. *IK* was knockdowned in human RPE cells, and wildtype-*IK* or mutant-*IK* (T485A; mutation of threonine to alanine cannot be phosphorylated) was re-transfected (Additional file 4: Fig. S4). Similar to the in vivo results, the *OFD1* mRNA level was downregulated in *IK*-knockdowned (KD) cells and rescued by wildtype-*IK*. In contrast, when the mutant-*IK* (T485A) was transfected into *IK*-KD cells, the *OFD1* mRNA expression still remained at low level without rescue. These results provide compelling evidence that *IK* is a transcriptional upstream regulator of *OFD1*. Previously, as IK was also known to play a role as a pre-mRNA splicing factor [19], we analyzed whether the downregulated *ofd1* expression in zebrafish *ik* mutants was induced by pre-mRNA splicing error. As a result, we observed that the *ofd1* pre-mRNA was normally spliced in *ik* mutants showing identical sequence with WT (Additional file 5). Furthermore, IK negatively regulates Aurora kinases, which are not only involved in the cell cycle but also play a role in cilia-associated signaling [16–18]. Thus, we investigated the expression of aurora kinases to examine the possibility that the downregulation of *ofd1* in *ik* mutants might be due to *aurora kinases*. In RT-PCR analysis, the mRNA level of *aurora kinase a* and *b* showed no difference between WT and *ik* mutants (Additional file 6: Fig. S5A). Moreover, knockdown of Aurora kinase A or B in RPE cells also did not affect to expression of OFD1 at protein level (Additional file 6: Fig. S5B). This implies that the downregulation of *odf1* in *ik* mutants occurs as a direct effect of *ik,* independent of *aurora kinases.*

### Ciliopathy phenotypes of *ik* mutants are rescued by co-injection of *ofd1*

**Fig. 5:**
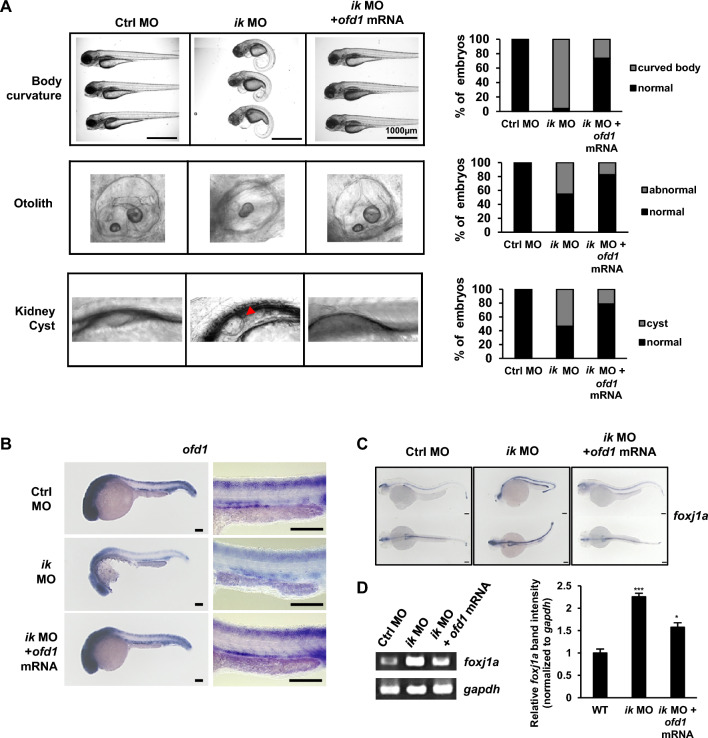
The ciliopathy phenotypes of *ik* mutants are rescued by *ofd1* co-injection (**A**) The lateral morphological view of control MO, *ik* MO, and *ik* MO/*ofd1* mRNA co-injected embryos (body curvature and otolith imaged at 2 dpf and pronephric cyst imaged at 3 dpf). Pronephric cyst in *ik* MO is indicated by red arrowhead. Stacked bar graph displays the phenotypic percentage of embryos. **B** WISH for *ofd1* in control MO, *ik* MO, and *ik* MO/*ofd1* mRNA co-injected embryos at 1 dpf. Scale bar, 100 μm. Enlarged view of the pronephros in control MO, *ik* MO, and *ik* MO/*ik* mRNA co-injected embryos at 1 dpf. Scale bar, 150 μm. **C** WISH for *foxj1a* in control MO, *ik* MO, and *ik* MO/*ofd1* mRNA co-injected embryos at 2.5 dpf. Scale bar, 100 μm (**D**) RT-PCR analysis of *foxj1a* mRNA expression in control MO, *ik* MO, and *ik* MO/*ofd1* mRNA co-injected embryos at 1.5 dpf. *gapdh* served as a normalization control. Relative band intensity of *foxj1a* normalized with respect to *gapdh* intensity is graphically represented using Image J software (in three individual experiments). *p < 0.05, ***p < 0.001

Next, to determine whether *ofd1* could rescue the ciliopathy phenotypes in *ik* mutants, we analyzed the phenotypes of *ik* morphants co-injected with *ofd1* mRNA (Fig. 5A). As expected, *ik* morphants co-injected with *ofd1* mRNA showed 73.7% rescued WT body curvature compared to that of *ik* morphants (4%) and normal otolith phenotype (82.6%) in contrast with that of *ik* morphants (54.8%). In addition, cyst formation observed in *ik* morphants by approximately 53% was reduced to 21.4% in *ik* morphants co-injected with *ofd1* mRNA. These results indicate that *ofd1* is a downstream target of *ik*. To exclude the off-target effect of co-injection of *ofd1* mRNA, we examined spatial *ofd1* expression in *ik* morphants and *ofd1* mRNA co-injected *ik* morphants using WISH experiments (Fig. 5B). The downregulated *ofd1* expression in *ik* morphants was enhanced by overexpressed *ofd1* mRNA, indicating no off-target effect in rescue experiments. Based on the rescued phenotype of kidney cyst formation in *ik* morphants co-injected with *ofd1* mRNA, we inferred that co-injection of *ofd1* mRNA into *ik* morphants would also moderate the impairment of kidney development or cyst formation and affect *foxj1a* expression. Thus, *foxj1a* expression was assessed by WISH experiment and RT-PCR (Fig. 5C, D). Consequently, the significantly upregulated *foxj1a* expression in the pronephros of *ik* morphants was alleviated by co-injection of *ofd1* mRNA in WISH (Fig. 5C). The mRNA level analysis of *foxj1a* in RT-PCR also demonstrated similar results (Fig. 5D). These results indicate that the ciliopathy phenotypes in the loss of *ik* result from the loss of *ofd1.*

### *ik* acts as the key regulator of *ofd1* and regulates proper ciliogenesis in vivo

**Fig. 6:**
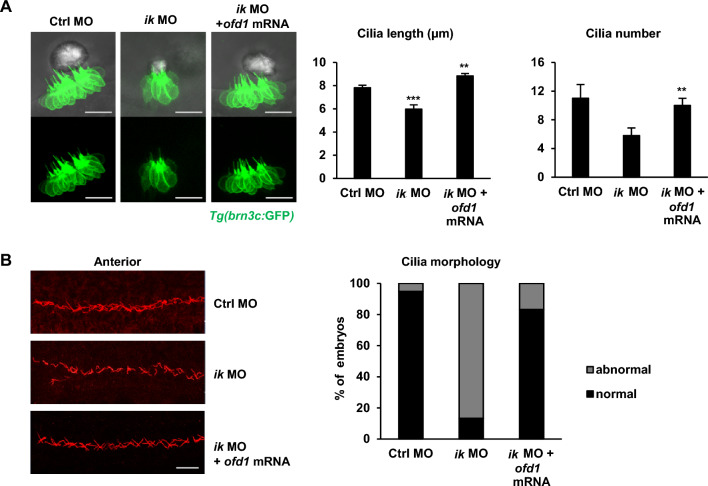
*ofd1* co-injection can rescue ciliary dysmorphology of *ik* morphants (**A**) Confocal microscopic images of control MO, *ik* MO, and *ik* MO/*ofd1* mRNA co-injected *Tg(brn3c:GFP)* embryos at 2 dpf. Scale bar, 20 µm. Bar graphs show quantification of ciliary length and number in the inner ears of control MO, *ik* MO, and *ik* MO/*ofd1* mRNA co-injected embryos at 2 dpf. **p < 0.01, ***p < 0.001 (**B**) Whole-mount immunostaining of pronephric cilia using anti-acetylated-α-tubulin (red) at 2 dpf control MO, *ik* MO, and *ik* MO/ *ofd1* mRNA co-injected embryos. Scale bar, 20 µm. Stacked bar graph displays the percentage of ciliary morphology of embryos in the anterior pronephric duct (percentage of abnormal ciliary morphology of embryos: control MO, 5.12% (37/39); *ik* MO, 86.6% (4/30); *ik* MO + *ofd1* mRNA, 16.6% (15/18)

Finally, to elucidate the correlation between *ik* and *ofd1* in ciliogenesis, we examined ciliary morphology of *ik* morphants co-injected with *ofd1* mRNA using the Tg(*brn3c*:GFP) zebrafish line. As expected, *ik* morphants co-injected with *ofd1* mRNA showed rescued cilia length and bundle numbers in hair cells compared to those of *ik* morphants. It also revealed comparable number of hair cells in the inner ear to that of the control morphant group (Fig. 6A). Similarly, in immunostaining for cilia in the pronephros, the randomly disorganized cilia morphology of *ik* morphants was predominantly restored by co-injection of *ofd1* mRNA (Fig. 6B). Taken together, *ik* contributes to proper ciliogenesis in vivo by regulating *ofd1*.

## Discussion

Ciliopathies emerge from functional and structural abnormalities of cilia [41]. Over the last two decades, the functions of genes related to ciliopathies have been revealed through genetic analysis of animal models and humans [42, 43]. Although 187 ciliopathy-associated genes have been characterized until recently, the roles of 241 candidate genes for ciliary function remain unclear [11]. The function of these candidate genes remains to be elucidated to understand the pathophysiology of ciliopathies. In this study, we showed that knockdown of *ik* induced ciliopathy phenotypes with disrupted ciliogenesis, which was accompanied by downregulation of ciliopathy-associated gene *ofd1*.

In general, failure to align the LR asymmetry sides of the cardiovascular system is often proposed as the main feature of ciliopathy [44]. However, *ik* mutants showed normally positioned LR loops in the cardiovascular system (Fig. 1C). We suggest two possible explanations for this phenomenon. First, this can be explained by the phenotypic expression of *ik* during embryogenesis. As embryonic transcripts are quiescent during early embryogenesis, maternally provided proteins and RNA directly contribute to early embryonic development [45–48]. And then, when the embryo enters the MZT period, transcriptional factors produced from the activated zygotic genome are governed by gene expression programs. We observed that *ik* transcript was detected at the early stage, but the expression level was significantly weaker than that at the zygotic period (Fig. 1A). Therefore, considering that LR asymmetry patterning arises in early somite stages [49], *ik* knockdown at an early stage would not affect phenotypes characterized during early embryogenesis. Second, *foxj1* is known to be required for LR body asymmetry during early embryogenesis [50]. Given the expression of *foxj1a* is significantly higher in *ik* mutants than in the WT embryos, *foxj1a* might perform complementary functions in the absence of *ik*. To understand the precise role of *ik* in ciliogenesis in vivo, further studies are needed to focus on the spatiotemporal effect of *ik* during embryogenesis.

Understanding ciliated cell types in specific tubular segments provides insights into the relationship between cilia and kidney development [37, 51]. Our study also demonstrates that *ik* mutants resulted in abnormal ciliary morphology in the pronephros, but interestingly, it was restricted only to proximal renal tubules and not distal tubules. Furthermore, we observed structurally abnormal development of PCT and PST segments in *ik* mutants (Fig. 3A). In nearly all vertebrates, two main ciliated cells exist: a single non-motile primary ciliated cell and MCCs [52]. Although ciliopathy syndromes linked to primary cilium defects have been extensively studied, studies on MCCs have been experimentally challenged because MCCs are located deep inside organs, and monitoring their behavior is difficult. However, zebrafish embryos have optical transparency; therefore, they have been used as a powerful model to study MCCs. In zebrafish, MCCs are present in several tissues, including the pronephros [53]. In the pronephros, MCCs are dispersed mainly in the PST region but are also adjacent within the PCT and DE segments. Given that disorganized ciliated cells were only observed in the PCT and PST in *ik* mutants, depletion of *ik* may specifically impair the proliferation or differentiation of MCCs in the proximal tubule. Interestingly, human clinical studies also showed similar results that patients with kidney diseases have abnormal MCCs with central microtubular pair (9 + 2 structure), whereas healthy people only contain non-motile primary cilia with 9 + 0 structure [54, 55], specifically in the proximal tubules not in the distal tubules [56]. These results suggest the possibility that abnormal differentiation of MCCs into proximal tubular cells would specifically affect kidney-related ciliopathy in vivo. Regarding the fact tha*t ik* mutants have phenotypes similar to those of human kidney patients, investigation of the role of IK using *ik* mutants will elucidate the molecular details of kidney ciliopathy in humans.

OFD type I syndrome is a rare ciliopathy disorder; however, more than seven million global patients have symptoms of renal cystic disease and other complicated disorders [57]. Because no effective pharmacological therapies for OFD syndrome is currently available, more biological and therapeutic approaches have been required. Here, we found that *ik* mutants had reduced *ofd1* mRNA expression. Because IK was also known as a pre-mRNA splicing factor in vitro, we additionally demonstrated whether there exists damage in pre-mRNA splicing of *ofd1* but downregulated *ofd1* in *ik* mutants was not from the pre-mRNA splicing error (Additional file 5). Based on these results, there are two possibilities to explain the cause of downregulated *ofd1* expression. First, it might be the result of a transcription factor defects of *ofd1* in *ik* mutants. Although we have clearly demonstrated in this study that *IK* directly contributes to expression of *OFD1* at transcription level through rescue experiments using *IK* mutant construct in vitro (Additional file 4: Fig. S4), the specific transcription factor of *OFD1* has not yet been identified. Therefore, it is necessary to investigate the specific transcription mechanism for *ofd1* gene. Second, we previously reported that knockdown of *ik* led genomic instability showing abnormal chromosome features in vitro [58]. Thus, loss of *ik* is also likely to induce genomic instability even in vivo leading to reduction of the *ofd1* mRNA expression. This possibility is also further supported by recent report showing mutation of SMU1, which is a reciprocal interactor of IK and functions as a unit by stabilizing each other, results in chromosomal abnormality with aberrant sister chromatid exchanges [59]. They referred that defects of these splicing factors could disrupt links between RNA metabolism and genomic recombination, thereby leading to abnormal gene expression. Therefore, it is worthwhile to explore the association between loss of *ik* and chromosomal instability to elucidate the regulatory mechanism of *ofd1*. In the underlying molecular mechanism mediated by the *ofd1*, *ofd1* inhibits autophagy by promoting the degradation of the unc-51-like kinase *ulk1* complex component *atg13* [60, 61]. Thus, the stabilized *ulk1* complex by the loss of *ofd1* enhances autophagy and causes OFD type I syndrome. As the *ofd1* expression was downregulated in loss of *ik* leading to ciliopathy phenotypes, further experiments are required to address whether loss of *ofd1* enhances autophagy in *ik* mutants. Considering that IK is well known to play a role in regulating mitotic cell cycle progression with mitotic kinase [16–18] and ciliogenesis is dependent on cell cycle progression under the control of autophagy, understanding the ciliogenesis–cell cycle–autophagy crosstalk between IK and OFD1 will provide another molecular aspect for understanding human ciliopathy.

## Conclusions

In conclusion, this study is the first to demonstrate the role of *ik* in ciliogenesis in vivo*.* In the absence of *ik*, zebrafish embryos display various ciliopathy phenotypes with abnormal ciliary morphology in ciliary tissues. We also address that *ofd1*, an essential gene for ciliogenesis*,* is a downstream target of *ik*. Elucidating the exact role of *ik* provides valuable insights into the understanding of ciliogenesis and ciliopathy in vivo.

## Materials and methods

### Zebrafish maintenance and husbandry

WT AB*, *Tg(brn3c:GFP),* and *Tg(bactin2:Arl13b-GFP)* zebrafish (*Danio rerio*) were used in this study. *ik* mutant zebrafish embryos have been previously described [20]. Embryos/larvae were raised at 28.5 °C and were natural spawning of adult zebrafish. Zebrafish were maintained in a tank rack system with automatic water recycling, water changes, and monitoring and adjustment of water parameters. Fish were kept under a 14 h light and 10 h dark cycles and were fed artemia (INVE aquaculture, Belgium) and pelleted dry food (Tetra, Blacksburg, VA, USA) thrice a day. Embryos were anesthetized with 0.2 mg/ml tricaine (3-aminobenzoic acid ethylester; Sigma-Aldrich, St. Louis, Missouri, USA) and embedded in methylcellulose. All zebrafish husbandry and experimental protocols complied with institutional guidelines and were approved by the local ethics board (Sookmyung Women’s University Animal Care and Use Committee, SMWU-IACUC-1712-036).

### Cell culture and transfection

The human retinal pigment epithelial cell line, hTERT-RPE1 were maintained in DMEM/F12 media supplemented with 10% FBS (Gibco, USA) at a humidified 37 °C, 5% CO_2_. IK siRNA transfection into RPE1 cells was performed using Lipofectamine RNAiMax Transfection Reagent (Invitrogen, USA) for 48 h according to the manufacturer's protocol with the siRNA oligonucleotides (5′- CUACCAAGGAGUUGAUCAA-3′) [58]. To knockdown of AURKA and AURKB, the siRNA of ON-TARGET plus SMART pool (Dharmacon) were used and transfection was performed according to the manufacturer's protocol. For rescue experiment, wildtype-full length human IK or mutant-IK T485A cloned into pcDNA 3.1 vector (Invitrogen, USA) was re-transfected using X-tremeGENE HP DNA Transfection Reagent (Roche, Germany) into siRNA transfected cells for 24 h according to the manufacturer's protocol.

### RT-PCR

Total RNA was extracted using TRIzol (Ambion, Foster City, California, USA), according to the manufacturer’s instructions. RNA concentration was measured using a NanoDrop (Thermo Fisher Scientific, Waltham, Massachusetts, USA), and 2 μg of RNA sample was reverse-transcribed into cDNA using a GoScript reverse transcription system kit (Promega, Madison, Wisconsin, USA).

Primer sequences for RT-PCR were designed using the National Center for Biotechnology Information (NCBI) primer-blast program (https://www.ncbi.nlm.nih.gov/tools/primer-blast/) (Additional file 7: Table S1).

### WISH

WISH was performed with standard protocols as previously described [62, 63]. Zebrafish embryos were fixed with 4% paraformaldehyde in phosphate-buffered saline (PBS) at 4 °C overnight and then rinsed with PBST (137 mM NaCl, 2.7 mM KCl, 10 mM Na2HPO4, 2 mM KH2PO4, 0.1% Tween 20) for rehydration. To increase the permeability of RNA probes, embryos were incubated with proteinase K (10 μg/ml), depending on their developmental stages. Embryos were refixed in 4% paraformaldehyde for 20 min at 25 °C and prehybridized in hybridization solution (HYB solution) containing 50% formamide, 5 × SSC (SSC: 3 M NaCl and 0.3 M sodium citrate, pH 7), 50 μg/ml heparin, 500 μg/ml RNA, 46 mM citric acid, pH 6, and 0.1% Tween 20 for at least 1 h at 65 °C. The HYB solution was removed, and the RNA probe was added to embryos at 65 °C and incubated overnight. The embryos were washed at 65 °C for 30 min once with 50% formamide in 2 × , 1 × , and 0.2 × SSC. For staining, embryos were incubated with blocking solution [2% sheep serum, 2 mg/ml bovine serum albumin (BSA) in PBST] for 2 h and then with anti-digoxigenin Fab fragment conjugated with alkaline phosphatase (Roche, Penzberg, Germany) at 1:5000 dilution at 4 °C overnight. Embryos were washed with PBST and visualized using BM Purple (Roche) as a substrate for alkaline phosphatase. After sufficient staining intensity was achieved, PBST was added to stop the reaction. Images were captured using a Leica DVM6 digital microscope (Leica, Germany) at Korea Basic Science Institute. Primers used for the RNA probe are provided in the Additional file 8: Table. S2, and *foxj1a* primers were designed as previously described [64].

### Immunofluorescence

For immunostaining of cilia in zebrafish embryos, embryos were fixed in 4% paraformaldehyde at 4 °C overnight and blocked with blocking solution (1 × PBS, 3% BSA, and 1% Triton X-100) at RT for 2 h. Zebrafish embryos were incubated with anti-acetylated-α-tubulin (Proteintech, CL594-66200, 1:500 dilution) at 4 °C overnight. Next, embryos were washed PBST (with 1% Triton X-100) for 30 min and incubated with Alexa Fluor 488-conjugated secondary antibodies (Sigma, 1:500 dilution) at 4 °C overnight. The nuclei of the fixed embryos were stained with 4', 6'-diamidino-2-phenylidole (DAPI). Images were acquired using a confocal microscope (LSM700, Zeiss, Oberkochen, Germany) at the Chronic and Metabolic Diseases Research Center of Sookmyung Women’s University.

### Immunoblotting

The Immunoblotting was performed as described previously [20]. The primary antibodies used for immunoblotting were as follows: rabbit polyclonal anti-OFD1 (Proteintech, 22851–1-AP), mouse monoclonal anti-β-actin (Santa Cruz, sc-47778), rabbit monoclonal p-AURKA Thr288 (Cell Signaling, #3079) and rabbit monoclonal p-AURKB Thr232 (Cell Signaling, #2914).

### TEM

Embryos were whole-mount fixed with Karnovsky’s fixative solution containing 2% glutaraldehyde and 2% paraformaldehyde overnight at 4 °C. After washing thrice with 0.05 M sodium cacodylate, they were postfixed with 1% aqueous osmium tetroxide. Then, embryos were stained en bloc using 0.5% aqueous uranyl acetate and were dehydrated through a graded series (30–100%) of ethanol, 20 min for each step. Embryos were incubated with progressively concentrated propylene oxide dissolved in ethanol and infiltrated with increasing concentrations of Spurr’s resin. The samples were embedded in Spurr’s resin, baked in an oven at 65 °C overnight, and sectioned using an ultramicrotome (EM UC7, Leica, Wetzlar, Germany). The sections were observed using a TEM (Talos L120C, FEI, Czech Republic) at the NICEM transmission electron microscope laboratory (Seoul National University, Seoul, Republic of Korea).

### Microinjection of morpholino oligonucleotide (MO) and mRNA

The MO sequence of *ik* was 5′-GGAGCCAGAGGATTAGAGTACACAT-3′, as previously described [20] and was obtained from GeneTools (Corvallis, OR, USA). For knockdown of *ik*, embryos at one-cell stage were injected with 4 ng of MO using a PV820 Pneumatic PicoPump (World Precision Instruments, Sarasota, Florida, USA) and reared in E3 medium (5 mM NaCl, 0.17 mM KCl, 0.33 mM CaCl_2_, 0.33 mM MgSO_4_, and 0.1% methylene blue) at 28.5 °C. Images were captured using a microscope (Olympus, Biotek Cytation 5) and a confocal microscope (LSM700, Zeiss, Oberkochen, Germany). For rescue experiments, full-length *ik* or *ofd1* mRNA was transcribed in vitro using a T7 mMESSAGE mMACHINE kit (Ambion, Foster City, California, USA) according to the manufacturer’s instructions. Embryos were then co-injected with 250 pg *ik* or *ofd1* mRNA with 4 ng of *ik* MO per egg.

### RNA-seq analysis

We used a previously analyzed RNA-seq dataset of *ik* mutants to evaluate differentially expressed genes (DEGs) [20]. In brief, genome-wide RNA-seq data of zebrafish embryos at 3 dpf were analyzed by eBiogen (Seoul, Korea). RNA with an absorbance ratio > 1.8 and integrity > 7.0 was confirmed. Fragments per kilobase of transcript per million mapped reads (FPKM) were used to determine gene expression. Upregulated or downregulated genes were identified using the ExDEGA program (EBIOGEN Inc., Seoul, Republic of Korea). Gene Ontology (GO) by the DAVID analysis was performed through Quick GO (https://www.ebi.ac.uk/QuickGO). Heatmap profiles of DEGs were prepared according to GO analysis using the Multiple Experiment Viewer software program v.4.9. A gene set representing > 1.5-fold change in *ik* KO embryos is presented by red color, and blue color represents < 1.5-fold change.

### Statistical analysis

The values in the graphs are presented as mean ± standard deviation (SD). Multiple comparisons within groups were performed using one-way analysis of variance (ANOVA), and differences between the means of individual groups were evaluated using Student’s *t*-tests. A *p*-value < 0.05 was considered as the threshold for significant differences (*p < 0.05, **p < 0.01, ***p < 0.001) and is provided in the figure legends.

### Supplementary Information

**Additional file 1: Figure S1.** The relative ik mRNA expression at different developmental stages. The ik mRNA expression at different developmental stages was quantified uisng qRT-PCR and normalized to β-actin. *p < 0.05, **p < 0.01, ***p < 0.001.

**Additional file 2: Figure S2.** Sections of whole-mount ISH probed with ik. (A) Dorsal (left) and lateral (right) view of 2-dpf embryo stained with the ik probe with planes of sections in follow panels as indicated (B) Section image of part of head with otic vesicle (arrow) of zebrafish embryo (C) Transverse cross section of the pronephric tubules (arrow).

**Additional file 3: Figure S3.** Large field-of-view images of cilia in the pronephros of ik mutants imaged by TEM. (A) Cross-sectional image of zebrafish pronephros. Motile cilia were observed in the sample (arrow). Scale bar, 200 nm (B) High magnification image of (A). Scale bar, 100 nm.

**Additional file 4: Figure S4.** The OFD1 mRNA expression in re-transfection of wildtype-IK or mutant-IK in RPE cells. The OFD1 mRNA expression in IK knock-downed human RPE cells which were re-transfected with cDNA encoding IK wild-type or T485A mutant.

**Additional file 5: **Sequencing results of ofd1 in ik KO embryos at 2.5 dpf.

**Additional file 6: Figure S5.** The expression analysis of aurora kinases. (A) The aurora kinase A (aurka) and B (aurkb) mRNA expression in WT embryos and ik mutants at 2 dpf (B) The OFD1 expression at the protein level in human RPE cells transfected with siAURKA or siAURKB for 48 h.

**Additional file 7: Table S1.** List of primer sequences used for RT-PCR analysis.

**Additional file 8: Table S2.** List of primer sequences used for WISH analysis.

## Data Availability

All datasets used and/or analyzed during the current study are available from the corresponding author on reasonable request.

## Abbreviations

MOs: Morpholino antisense oligonucleotides
MZT: Maternal to zygotic transition
OFD1: Oral-facial-digital 1
PCR: Proximal convoluted tubule
TEM: Transmission electron microscope
