# Supplementary material for: IK is essentially involved in ciliogenesis as an upstream regulator of oral-facial-digital syndrome ciliopathy gene, ofd1
Source: Cell Biosci. 2023 Oct 28;13:195. doi: 10.1186/s13578-023-01146-9 (PMC10612314; doi:10.1186/s13578-023-01146-9)
Supplement: Supplementary file 1 — Additional file 1: Figure S1. The relative ik mRNA expression at different developmental stages. The ik mRNA expression at different developmental stages was quantified uisng qRT-PCR and normalized to β-actin. *p < 0.05, **p < 0.01, ***p < 0.001. [file 13578_2023_1146_MOESM1_ESM.docx]

Additional file for

**IK is essentially involved in ciliogenesis as an upstream regulator of oral-facial-digital ciliopathy syndrome gene, *ofd1***

Hye In Ka^1,2^, Mina Cho^1^, Seung-Hae Kwon^3^, Se Hwan Mun^1,2^, Sora Han^1^, Min Jung Kim^1,4,^* and Young Yang^1,2,4,^*

^1^ Research Institute of Women’s Health, Sookmyung Women's University, Seoul 04312, South Korea

^2^ Chronic and Metabolic Diseases Research Center, Sookmyung Women's University, Seoul 04312, South Korea

^3^ Seoul Center, Korea Basic Science Institute, Seoul 02841, South Korea

^4^ Department of Biological Sciences, Sookmyung Women’s University, Seoul 04312, South Korea

* Correspondence: Min Jung Kim (minkim@sookmyung.ac.kr) and Young Yang (yyang@sookmyung.ac.kr)

**This PDF file includes:**

Additional File 1: Figures S1

Additional File 2 : Figures S2

Additional File 3 : Figures S3

Additional File 4: Figures S4

Additional File 5

Additional File 6: Figures S5

Additional File 7: Table S1

Additional File 8: Table S2

**Additional File 1**

**Figure S1. The relative *ik* mRNA expression at different developmental stages**

The *ik* mRNA expression at different developmental stages was quantified uisng qRT-PCR and normalized to β-actin. **p* < 0.05, ***p* < 0.01, ****p* < 0.001
