# Supplementary material for: IK is essentially involved in ciliogenesis as an upstream regulator of oral-facial-digital syndrome ciliopathy gene, ofd1
Source: Cell Biosci. 2023 Oct 28;13:195. doi: 10.1186/s13578-023-01146-9 (PMC10612314; doi:10.1186/s13578-023-01146-9)
Supplement: Supplementary file 2 — Additional file 2: Figure S2. Sections of whole-mount ISH probed with ik. (A) Dorsal (left) and lateral (right) view of 2-dpf embryo stained with the ik probe with planes of sections in follow panels as indicated (B) Section image of part of head with otic vesicle (arrow) of zebrafish embryo (C) Transverse cross section of the pronephric tubules (arrow). [file 13578_2023_1146_MOESM2_ESM.docx]

**Additional File 2**

**
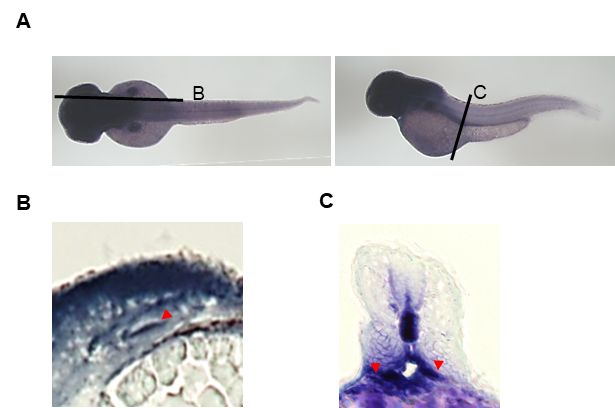
**

**Figure S2. Sections of whole-mount ISH probed with *ik.*** (A) Dorsal (left) and lateral (right) view of 2-dpf embryo stained with the *ik* probe with planes of sections in follow panels as indicated (B) Section image of part of head with otic vesicle (arrow) of zebrafish embryo (C) Transverse cross section of the pronephric tubules (arrow).
