# Supplementary material for: IK is essentially involved in ciliogenesis as an upstream regulator of oral-facial-digital syndrome ciliopathy gene, ofd1
Source: Cell Biosci. 2023 Oct 28;13:195. doi: 10.1186/s13578-023-01146-9 (PMC10612314; doi:10.1186/s13578-023-01146-9)
Supplement: Supplementary file 3 — Additional file 3: Figure S3. Large field-of-view images of cilia in the pronephros of ik mutants imaged by TEM. (A) Cross-sectional image of zebrafish pronephros. Motile cilia were observed in the sample (arrow). Scale bar, 200 nm (B) High magnification image of (A). Scale bar, 100 nm. [file 13578_2023_1146_MOESM3_ESM.docx]

**Additional File 3**

**
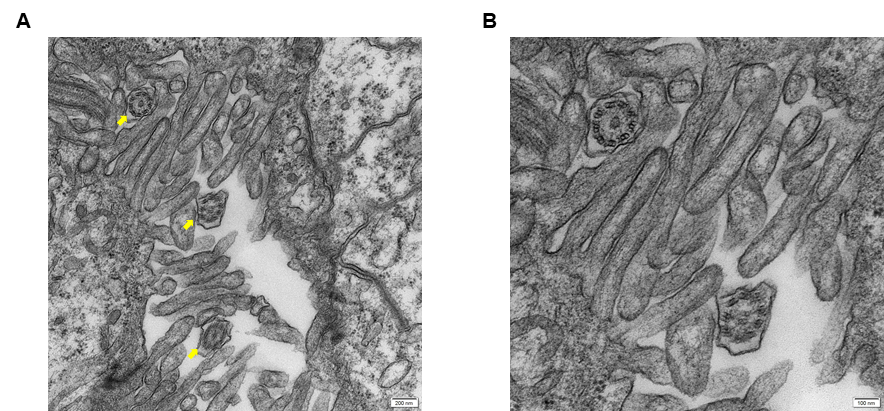
**

**Figure S3. Large field-of-view images of cilia in the pronephros of *ik* mutants imaged by TEM.** (A) Cross-sectional image of zebrafish pronephros. Motile cilia were observed in the sample (arrow). Scale bar, 200 nm (B) High magnification image of (A). Scale bar, 100 nm.
