# Supplementary material for: IK is essentially involved in ciliogenesis as an upstream regulator of oral-facial-digital syndrome ciliopathy gene, ofd1
Source: Cell Biosci. 2023 Oct 28;13:195. doi: 10.1186/s13578-023-01146-9 (PMC10612314; doi:10.1186/s13578-023-01146-9)
Supplement: Supplementary file 4 — Additional file 4: Figure S4. The OFD1 mRNA expression in re-transfection of wildtype-IK or mutant-IK in RPE cells. The OFD1 mRNA expression in IK knock-downed human RPE cells which were re-transfected with cDNA encoding IK wild-type or T485A mutant. [file 13578_2023_1146_MOESM4_ESM.docx]

**Additional File 4**


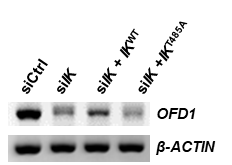


**Figure S4.** **The *OFD1* mRNA expression in re-transfection of wildtype-*IK* or mutant-*IK* in RPE cells.** The *OFD1* mRNA expression in *IK* knock-downed human RPE cells which were re-transfected with cDNA encoding *IK* wild-type or T485A mutant.
