# Supplementary material for: IK is essentially involved in ciliogenesis as an upstream regulator of oral-facial-digital syndrome ciliopathy gene, ofd1
Source: Cell Biosci. 2023 Oct 28;13:195. doi: 10.1186/s13578-023-01146-9 (PMC10612314; doi:10.1186/s13578-023-01146-9)

**Additional File 5. Sequencing results of *ofd1* in *ik* KO embryos at 2.5 dpf**

To analyze the full sequence of *ofd1* in *ik* KO embryos at 2.5 dpf, full length *ofd1* PCR products were cloned to pBHA-T vector and verified through sanger sequencing (Cosmogenetech, Seoul, Korea).

**● Sequencing results of *ofd1* exon1-exon7 in *ik* KO:**

CCTAACATATGTGTGTTTGTTTGGGGGCGGGGAGAGAGCGAAGAAGCTCTATCAGACCTTCAAGAGCCGCGGGCTGCTGGACACTCTGAAGACACGGCTGCGGAATCAGCTGATCCAGGAGCTGCAGGCGCCGGTGCGCAGAGGAGAATCTGCTTCCAGACGCTCTGCAGATCACACTGACTCTGTGTTAGTCTCTGCATGCAACAGTGTGGTGGTCGATCACCTGCGCAGCGCTGGCTATGAGTACACACTGTCTGTCTTCCAGCCCGAATGCGGACTGAGCAAAGACAAGGTGTTATCAAGTCGTGATGTTCTGCAGATCGTGAAAATCAGCCCTCATATGCCCTTATACAAATCTCTGGTTTCAAATATCCAGAGGGGACAGTCAGGTTTTCTGAAGAGTCTGTTGATGGAGCTGACGGACCACAGTGTTTACAGTGACTGCAGTGATAATTCAACTCAAACCACCTCTATAGCAGCGCATAAGGAGTCTCTGGTAGAGAAGATGCAGCTGATTGATGAGGAGTATGAAGTGCTGCGGCACAGAGGCGACCGCTGGGCGTCAGTGGAAGCCAAACTGGCCGAATACAGGAAAGAGATCCAGGAACAAGCACAGATCGAGCTCAACGCAAAGCTCCAGCACTTCATGGATGTGGAGATAGCGAAGGTAAAGCAGGAAGAGAAAGAAAGATCGAGGAAAGAAATCCTGGAGCTCAGACGAGACATGGAGAAAACATATGAGCTGAAGTCTGAAGCTCTGATCAGTCGAGAGAAAAACGCCATCGAGCGCTTGCAGAAACACCAGGAGATTGAAGAAAAGGACATTTATGCTCAAAGACAAGCCGTGCTGAGGGAAATCGAGTCTGTGCGGAGCCGAGAGATGGAGCTGAGGCAGAGGATGGAGGCCTTTGACAAGAGCTGTGCGCTGCACGAGGAGAAGGTGAAGACCATGGAGGATCTCCTACGGAGGAGAGAACTGTCTGCGAAGACCATGGAGGATTCCTTTGAGCAGAAACTGAAGAGCGAGTTACTGAAATATCAGCTGGAACTGAAGGAGGAGAATATGAAGAGAACAGAGAAACTGACAGA


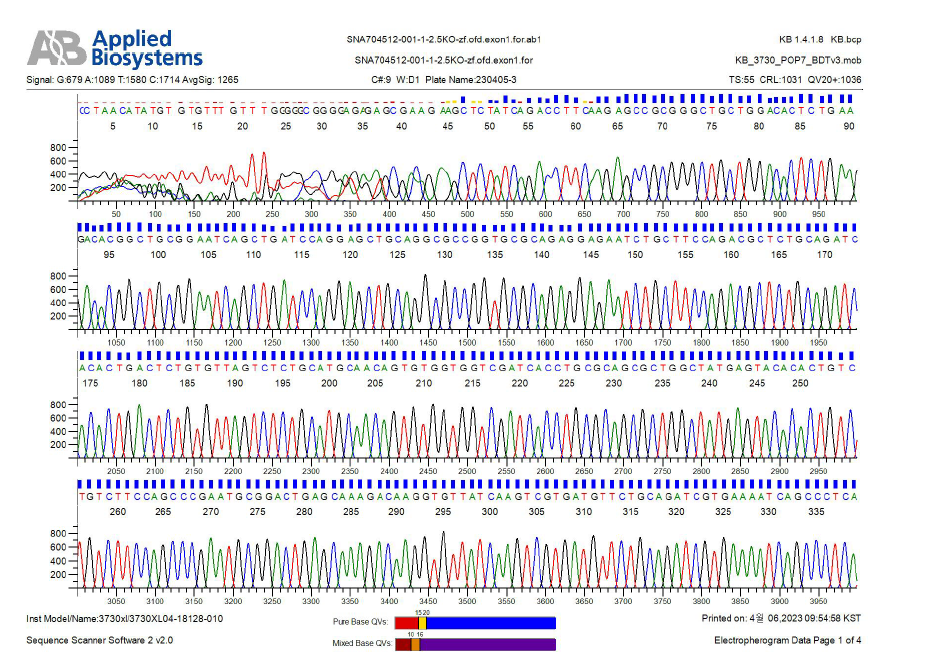

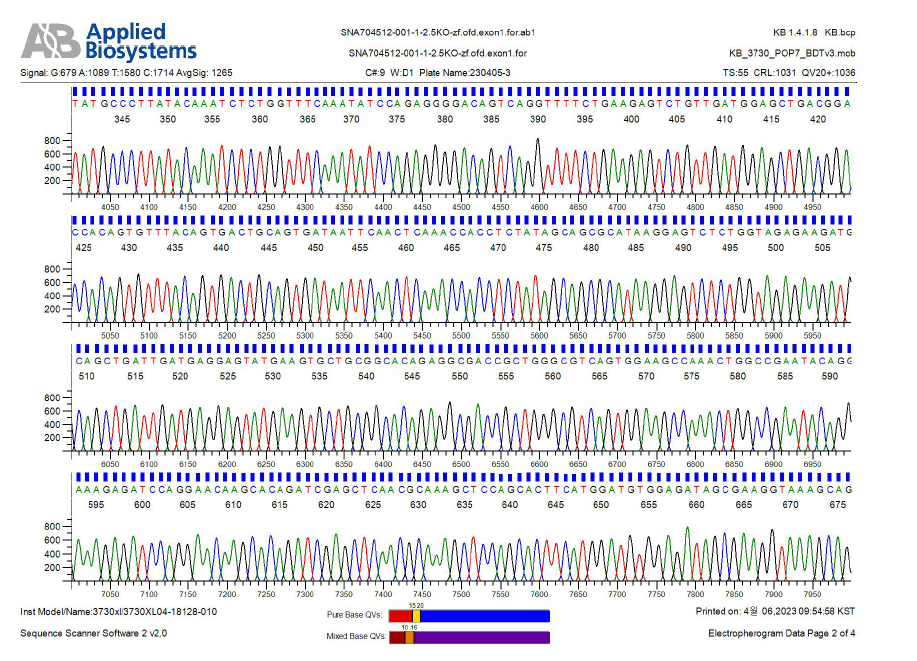

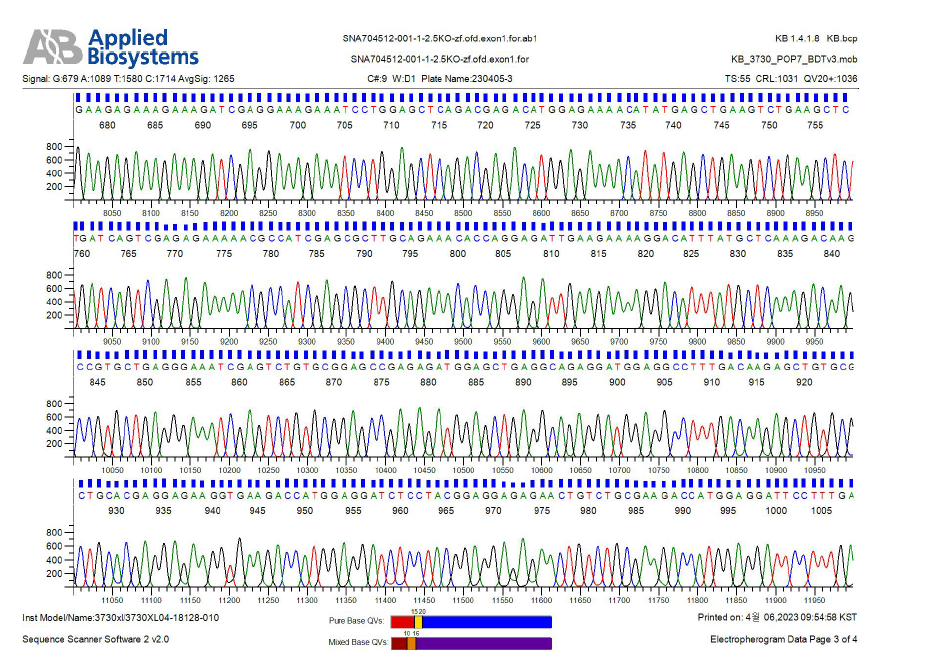

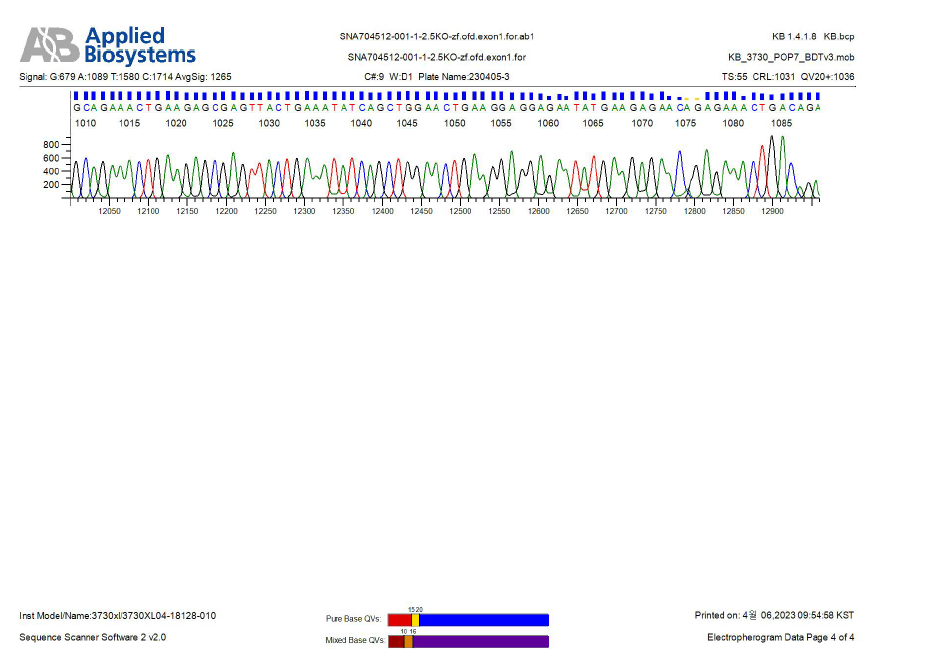


**● Sequencing results of *ofd1* exon5-exon13 in *ik* KO:**

TAACAGTAAGGCACCCGGTGGTATCCAGGTGACTGCAGTGATAATTCAACTCAAACCACCTCTATAGCAGCGCATAAGGAGTCTCTGGTAGAGAAGATGCAGCTGATTGATGAGGAGTATGAAGTGCTGCGGCACAGAGGCGACCGCTGGGCGTCAGTGGAAGCCAAACTGGCCGAATACAGGAAAGAGATCCAGGAACAAGCACAGATCGAGCTCAACGCAAAGCTCCAGCACTTCATGGATGTGGAGATAGCGAAGGTAAAGCAGGAAGAGAAAGAAAGATCGAGGAAAGAAATCCTGGAGCTCAGACGAGACATGGAGAAAACATATGAGCTGAAGTCTGAAGCTCTGATCAGTCGAGAGAAAAACGCCATCGAGCGCTTGCAGAAACACCAGGAGATTGAAGAAAAGGACATTTATGCTCAAAGACAAGCCGTGCTGAGGGAAATCGAGTCTGTGCGGAGCCGAGAGATGGAGCTGAGGCAGAGGATGGAGGCCTTTGACAAGAGCTGTGCGCTGCACGAGGAGAAGGTGAAGACCATGGAGGATCTCCTACGGAGGAGAGAACTGTCTGCGAAGACCATGGAGGATTCCTTTGAGCAGAAACTGAAGAGCGAGTTACTGAAATATCAGCTGGAACTGAAGGAGGAGAATATGAAGAGAACAGAGAAACTGACAGAAAATGAGGAGAGGATTAGAGCAGAAGCAGCTCGTCTGCAGAAAGAAGCTGCTGTTATTGAAGCAAAGACAGAAGATTATGAGAGAAAATCATCAGAGGTCAAACAACTGCTGATGGAGCTGGAGTCGTCCCGATCGCAGGCGTCTCTCCTGAAGCAACAGAAGGAGCTGCTGAGAGAACAACTGGAGAACATGAGGGATTATCCAGAGCTGAAGAAACACACGCTGGAGCTGCAGACACGCATCACCCTGCTGAAACAACAGCTGGAGGAGAAACAACAACACAACCAGAGGCTGACACAAGAGCTCAGCGCGCCGTCACATGAGCATCTGATGCTGCAGGCGGAGCTTCGGAGACTGGAGGCGGAGCATAAACTGCAGAAGGAGG


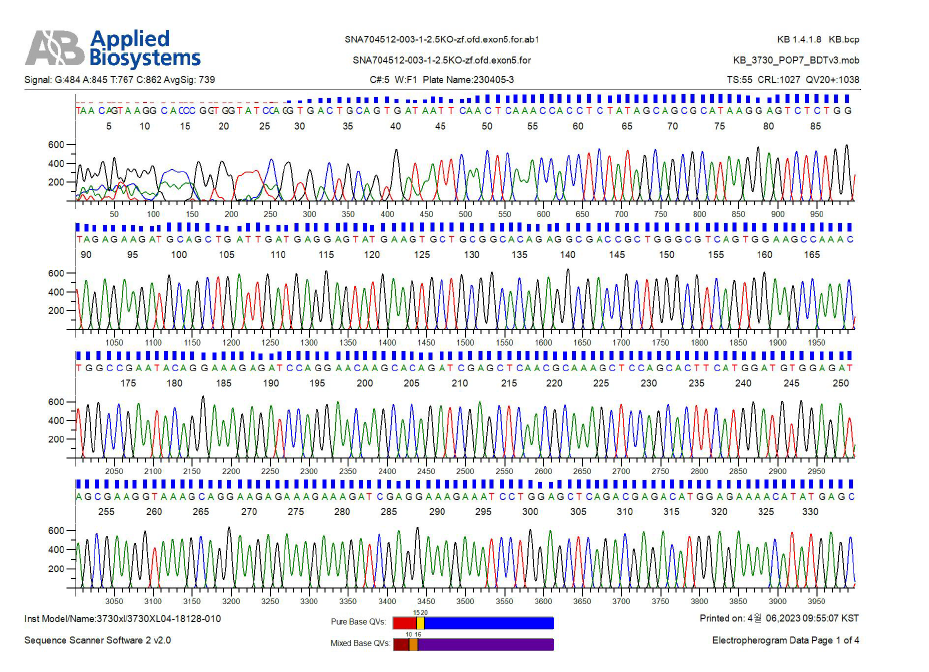

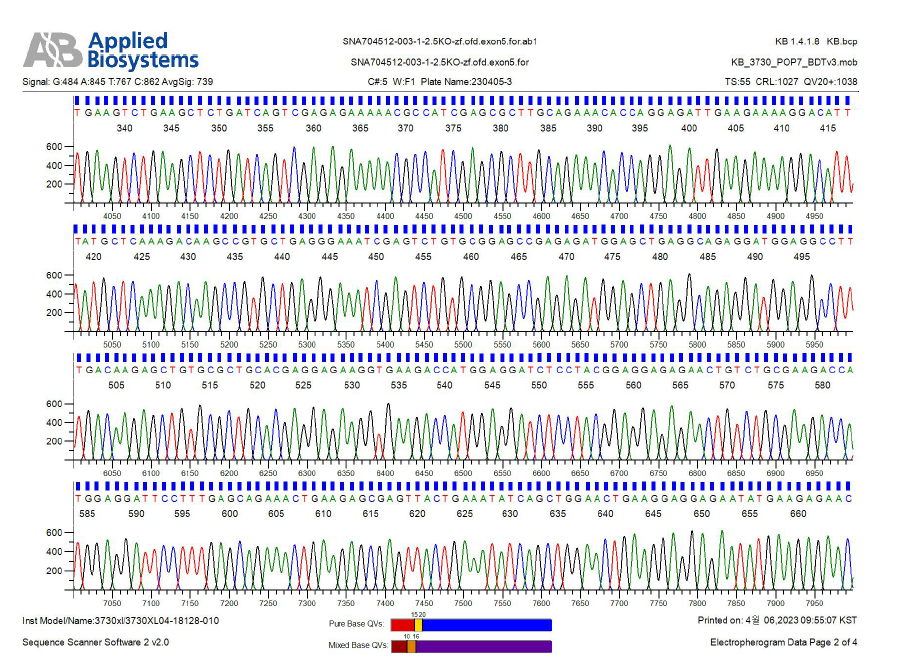

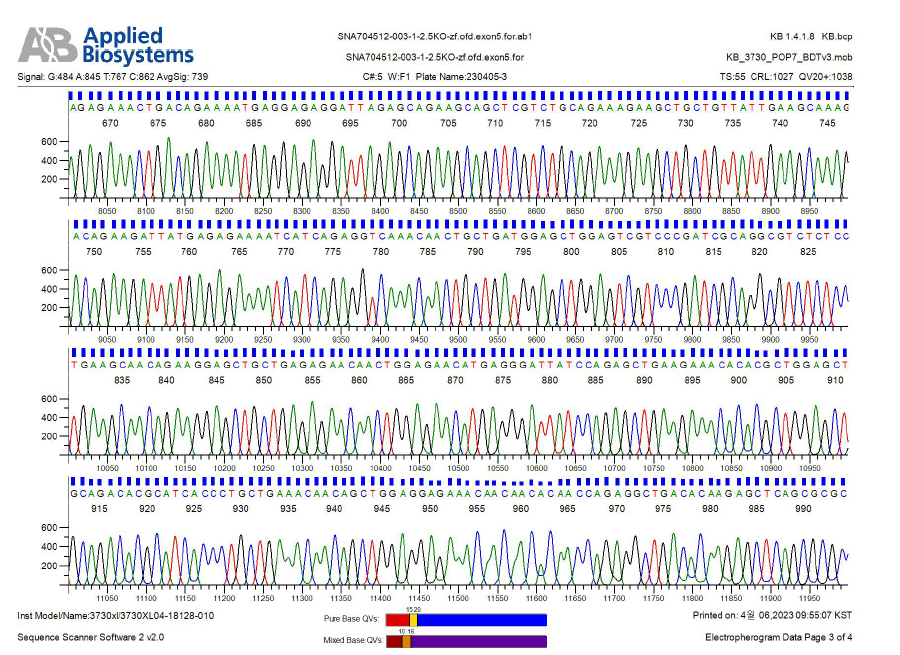

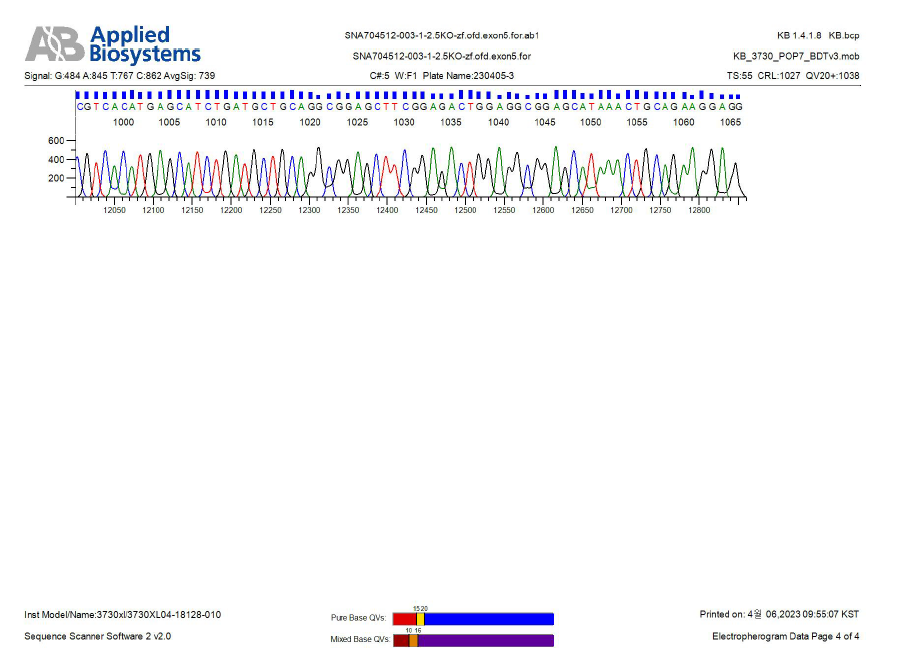


**● Sequencing results of *ofd1* exon11-exon15 in *ik* KO:**

GTTAGATATCGAGAACTGACAGAAATGAGGAGAGGATTAGAGCAGAAGCAGCTCGTCTGCAGAAAGAAGCTGCTGTTATTGAAGCAAAGACAGAAGATTATGAGAGAAAATCATCAGAGGTCAAACAACTGCTGATGGAGCTGGAGTCGTCCCGATCGCAGGCGTCTCTCCTGAAGCAACAGAAGGAGCTGCTGAGAGAACAACTGGAGAACATGAGGGATTATCCAGAGCTGAAGAAACACACGCTGGAGCTGCAGACACGCATCACCCTGCTGAAACAACAGCTGGAGGAGAAACAACAACACAACCAGAGGCTGACACAAGAGCTCAGCGCGCCGTCACATGAGCATCTGATGCTGCAGGCGGAGCTTCGGAGACTGGAGGCGGAGCATAAACTGCAGAAGGAGGAGTTGGAGACGCAGAAGAATGTCTTACACACACAACTGCAGCACGAGGTCCAACAGTGTGCGCTGTTGAAGGCTCAGCTGATGGAGTGTGAAGAACGCACTAAATGGATGAACACACACACTGAAGAGCTCAAACTACAGCTACAGCAGACTCAGCAAGCTCTAGAAAACGAGATCCTCCGAAACCCCAAGCCTTCTCTGGTAGACCGATCTGTTCTCAATCTGCACCCTGATAAACTCCTTCCTCCTGATATATACATAGACACTGAACTCCTCAGAAACACCAGAGCCGCTGCTGACGGTAATGTATCTGAAGCAGGACTGGCTCTCAGGGGCCCGAAATCTCGCACTGTGGCCCCTGAACATGATTCTGACATGGTTATCAGTGCTTTATCCCGCATTCGAGAGCTGGAGCAGGAGGCGGAGCGGCTAGAGGAGGCGTACAGGAGTCACCAGCAGAGGGCGCTGTCTGCTGAAGATCCCACACTGCACAGAGGCAGCCAGAACTACAGCAGAGACACAGCAGCGCAGCAGCACAGAGTCATATCCCGCAGTCCTATTTTTGCGGGACGTCCCATTGAGGAGGAGCATGAGGAGTTCAGTAGGACTCCTTCTCCAGCAGAGA


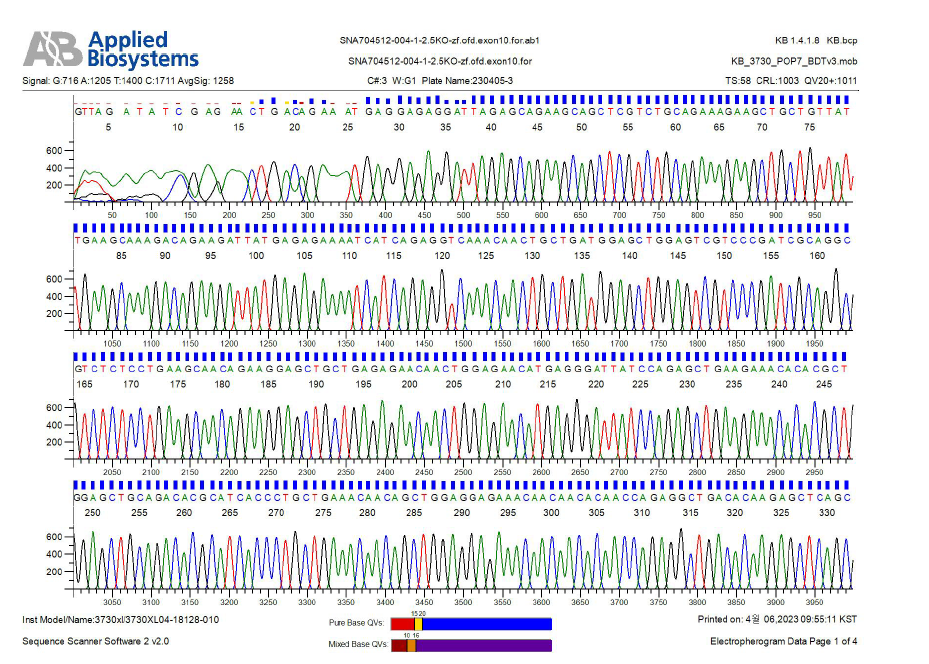

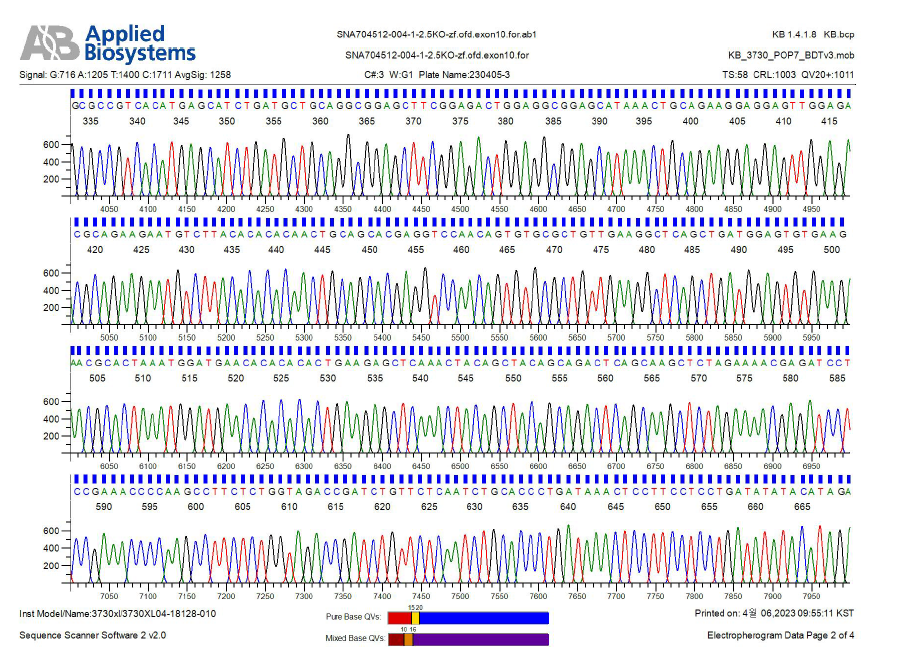


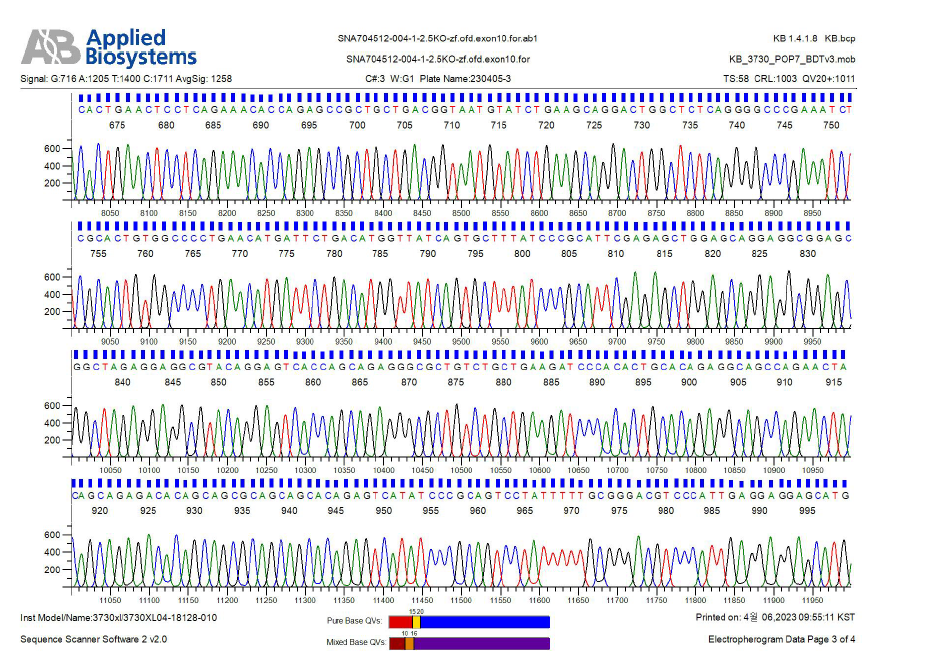

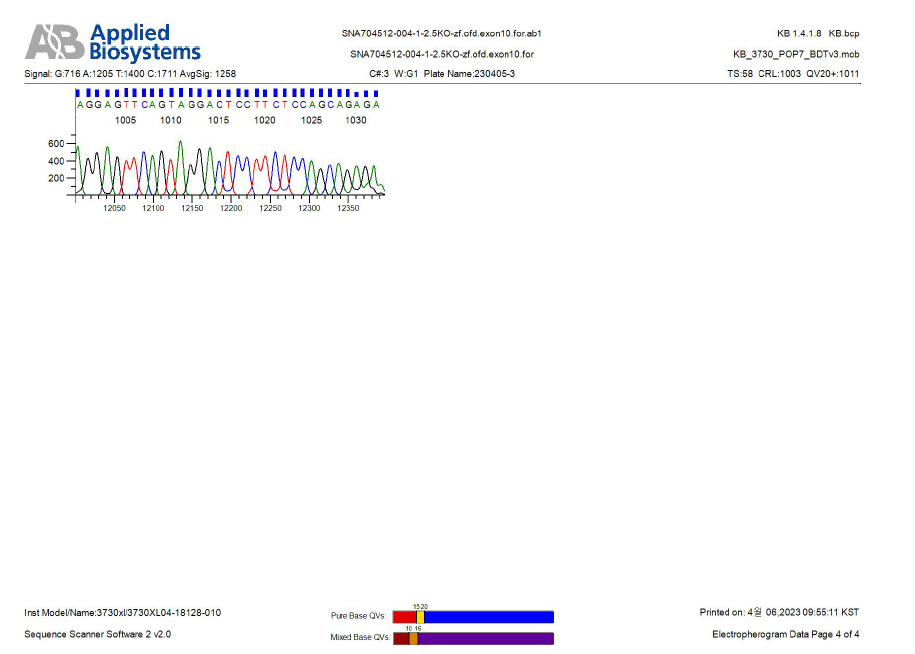


**● Sequencing results of *ofd1* exon16-exon23 in *ik* KO:** ACACGGCCGTATCAATATTTTCCCTTTGGGGAAGTCCATTGAGGAGGAGCATGAAGGAGTTCAGTAGGACTCCTTCTCCAGCAGAGCGACTCGCCTCTCCTCCTGCCAGACGTCTGTCCTCCACACCACAGTCTGCTTCCAGGAGCAAACGCCGAGCAGATGAGGAGCATGATGAACACTCTTTAAATACAGAGTGTGTGAAAGTATCCTCTAACACGGACAGACCAGCGCTGATGTTTCCAGAAAGACTCATTTCACCCATTCCTGCTGAGGAACTGAGCTCCTCCATCAGTCCCAGCAGCCCCGTCATGAAGAGCACCACACGACACACACAAAGTCCAGCCAAACTGCAGGAAATCCTCTTGTCCAGCTCCTCTCAGGAATCCTCACCGCAGCCAGAGAAAATCACACTGCACGACCTGACGGAGCCCATACAGATGGTATCTGCAGATCAGCCGTGTCTCCTTCAGGATTGTGAGCCGGAGCTCCAGCAGGATCATCCAGATGTACAAATATCATCATCATCATCATCATCATCATCATCACAGCGTGAGGAAGAGCAGCAGAGAGAGACACACACACTCCAGCAGCAGCCGCATGAGGAACAAACACAAGAGCAGCGTGATGATGCTGGGGGTCATGTGACTTCAGCGGCCTCACCGACTGGAGGAGCAGAAGAGGCCAATCCGCTGCAGAGATACATGCAGATGCTGATGCAGGACAAACAGCAGGAACAGTCTCCAAATAAAGAGTCATCAGGGAGTCACGAGGAGAATCTTCAGTCTGAGAATCACGAGCACAGCGCCGGCGTGATTTCACACGATGAAGCCGACGATGATTTCTGGAAGCTTAGTATTGGATCTGCAGAATTCCAGCACACTGGCGGCCGTTACTAGTGGATCCGAGCTCGGTACCAAGCTTGATGCATAGCTTGAGTATTCTATAGTGTCACCTAAATAGCTTGGCGTAATCATGGTCATAGCTGTTTCCTGTGTGAAATTGTTATCCGCTCACAATTCCACACAACATACGAGCCGGAAGCATAAAGTGTAAAGCCTGGGGTGCCTAATGAGTGAGCTAACTCACATTAATCACCTGTAAGTCGGACGAATTCGGCGCTCTTCCGCTTCCTCGCTCACTGACTCGCTGCGCTCGGTCGTTCGGCTGCGGCGAAGCGTATCAGCTCA


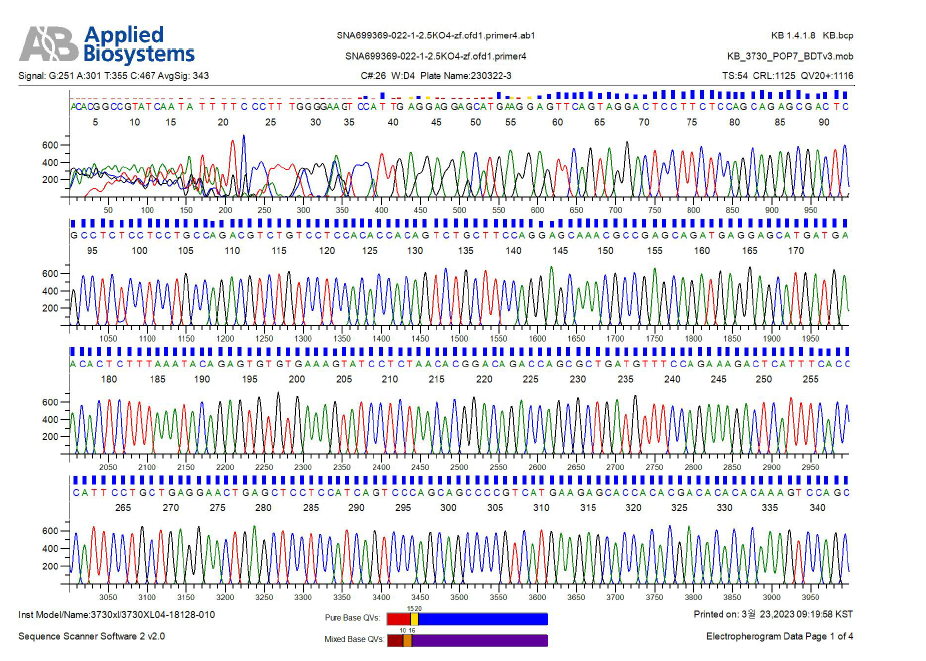


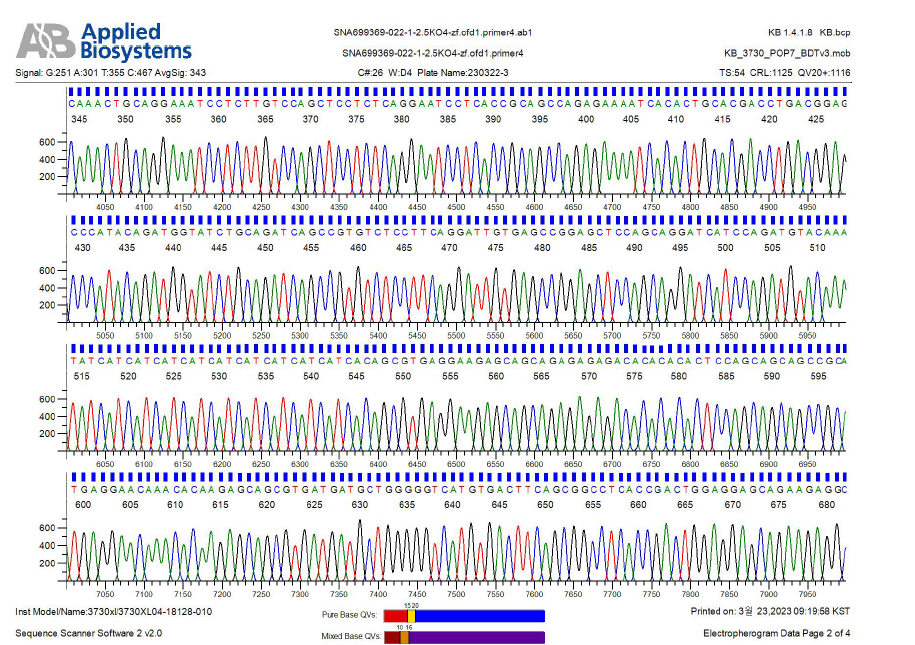


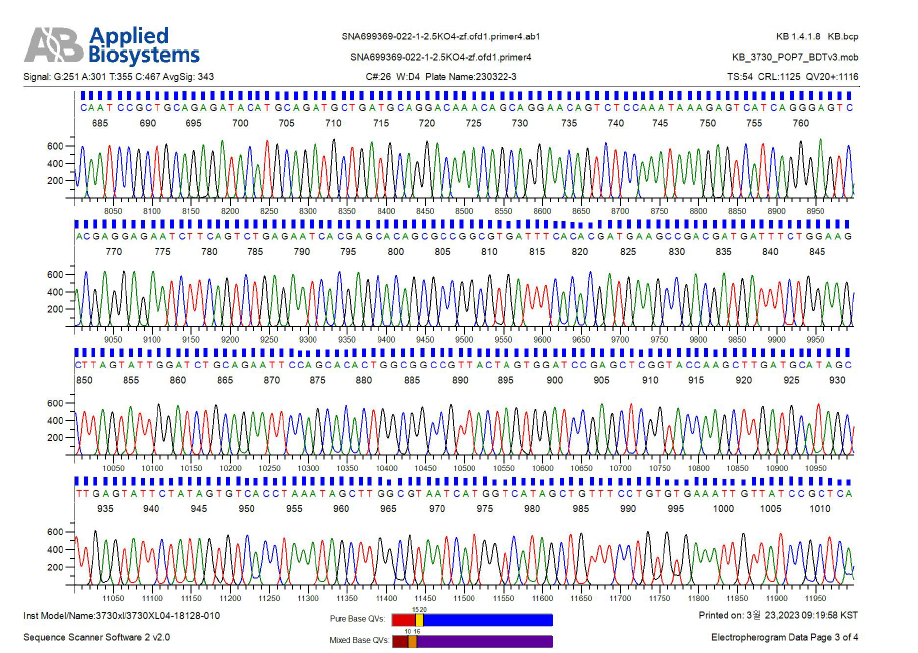

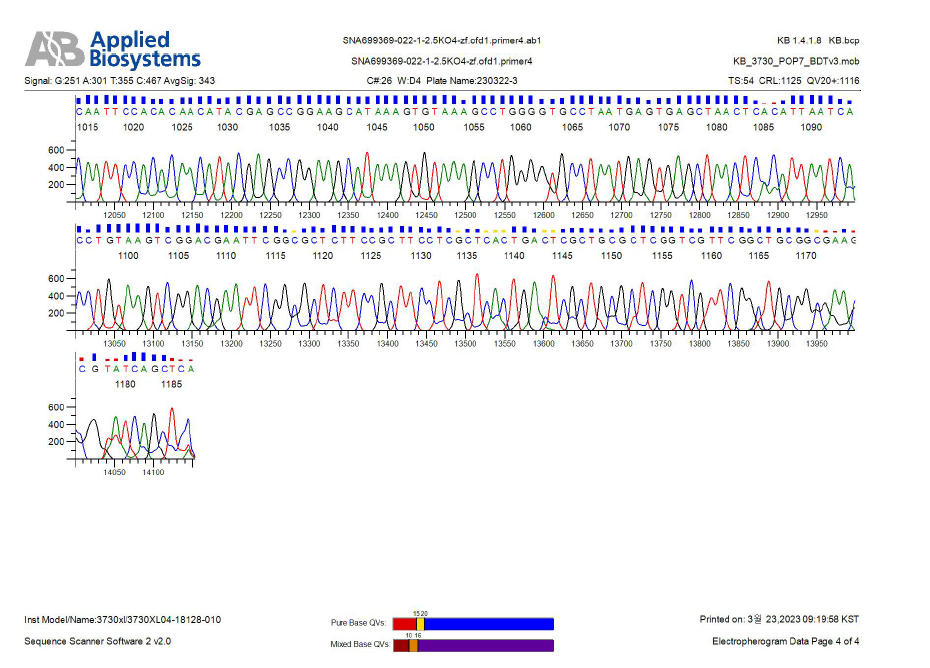

Supplement: Supplementary file 5 — Additional file 5: Sequencing results of ofd1 in ik KO embryos at 2.5 dpf. [file 13578_2023_1146_MOESM5_ESM.docx]
