# Supplementary material for: IK is essentially involved in ciliogenesis as an upstream regulator of oral-facial-digital syndrome ciliopathy gene, ofd1
Source: Cell Biosci. 2023 Oct 28;13:195. doi: 10.1186/s13578-023-01146-9 (PMC10612314; doi:10.1186/s13578-023-01146-9)
Supplement: Supplementary file 6 — Additional file 6: Figure S5. The expression analysis of aurora kinases. (A) The aurora kinase A (aurka) and B (aurkb) mRNA expression in WT embryos and ik mutants at 2 dpf (B) The OFD1 expression at the protein level in human RPE cells transfected with siAURKA or siAURKB for 48 h. [file 13578_2023_1146_MOESM6_ESM.docx]

**Additional File 6**


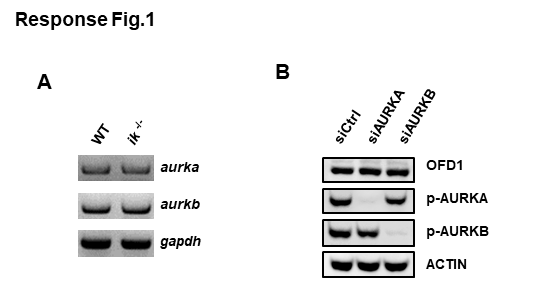


**Figure S5. The expression analysis of aurora kinases.** (A) The *aurora kinase A* (*aurka*) and *B* (*aurkb*) mRNA expression in WT embryos and *ik* mutants at 2 dpf (B) The OFD1 expression at the protein level in human RPE cells transfected with siAURKA or siAURKB for 48 h
