# Supplementary material for: IK is essentially involved in ciliogenesis as an upstream regulator of oral-facial-digital syndrome ciliopathy gene, ofd1
Source: Cell Biosci. 2023 Oct 28;13:195. doi: 10.1186/s13578-023-01146-9 (PMC10612314; doi:10.1186/s13578-023-01146-9)
Supplement: Supplementary file 7 — Additional file 7: Table S1. List of primer sequences used for RT-PCR analysis. [file 13578_2023_1146_MOESM7_ESM.docx]

**Additional File 7: Table S1. List of primer sequences used for RT-PCR analysis**

| **Name of Genes** |  | **Primer sequences (5’-3’)** |
| --- | --- | --- |
| Zebrafish *ik* | Forward | TACGCTGAATGCTACCCTGC |
|  | Reverse | TGAGTGTCAAAGTCCCAGCG |
| Zebrafish *gapdh* | Forward | GTATGACTCCACCCACGGC |
|  | Reverse | ACAATGGTCATGCTGGAGGG |
| Zebrafish *slc4a4* | Forward | GATCGGCCCAAACTGCTTCT |
|  | Reverse | GCTCCTGCTCCATCATGTCC |
| Zebrafish *slc13a1* | Forward | GCATCTCCAACCCAGCTCTT |
|  | Reverse | CCGAGGTACATCCAGTGCAG |
| Zebrafish *slc12a3* | Forward | GACCGGAGGGCAGTTTCTTT |
|  | Reverse | GAGGAAAGGGTGGCTCCAAA |
| Zebrafish *foxj1a* | Forward | AGTCCAACCCGCACATCAAG |
|  | Reverse | GTTCATCCTGAGTCGGTGCT |
| Zebrafish *ofd1*  Zebrafish *aurka*  Zebrafish *aurkb*  Human *OFD1*  Human *β-ACTIN* | Forward | CCTGAAGCAACAGAAGGAGC |
|  | Reverse  Forward  Reverse  Forward  Reverse  Forward  Reverse  Forward  Reverse | TGCTTGCTGAGTCTGCTGTA  CGGATGCTCTGAGATACTGCC  ATGGGGAGTCTGTGCATTGG  ACTGGATTA AACAGCCGCCA  AAACACACGGGTGTCGTCAT  GAACCGCGAAGAAAGGAAGC  GCTGGTTTCGAAGTTGTGTCT  GTGGGCCGCCCTAGGCACCA  CTCTTTGATGTCACGCACGA |
