# Supplementary material for: IK is essentially involved in ciliogenesis as an upstream regulator of oral-facial-digital syndrome ciliopathy gene, ofd1
Source: Cell Biosci. 2023 Oct 28;13:195. doi: 10.1186/s13578-023-01146-9 (PMC10612314; doi:10.1186/s13578-023-01146-9)
Supplement: Supplementary file 8 — Additional file 8: Table S2. List of primer sequences used for WISH analysis. [file 13578_2023_1146_MOESM8_ESM.docx]

**Additional File 8: Table S2. List of primer sequences used for WISH analysis**

| **Name of Genes** |  | **Primer sequences (5'–3')** |
| --- | --- | --- |
| Zebrafish *ik* | Forward | TTTTTGCAGGATCCATGCCTGAACGCGAAA |
|  | Reverse | TTCTAGAGGCTCGAGCTAATATTTTGGCCGCTTGA |
| Zebrafish *cmlc2* | Forward | GAGGTAATACGACTCACTATAGGGATGGCTAGTAAG AAA GCC GCG |
|  | Reverse | GAGGAATTAACCCTCACTAAAGGGTCAAGATTCCTCTTT TTCATC |
| Zebrafish *slc4a4* | Forward | GAGGTAATACGACTCACTATAGGGATGAGCGCCGGC AAG AGG GTG |
|  | Reverse | GAGGAATTAACCCTCACTAAAGGGCTCTTCGAACTTGAT CCACC |
| Zebrafish *slc13a1* | Forward | GAGGTAATACGACTCACTATAGGGATGAGGCGGCTT AGATGTTCT |
|  | Reverse | GAGGAATTAACCCTCACTAAAGGGGTTGCTCAACCA CATAGAGAGAA |
| Zebrafish *slc12a3* | Forward | GAGGTAATACGACTCACTATAGGGATGGAGAATCCA GGCTTCCAG |
|  | Reverse | GAGGAATTAACCCTCACTAAAGGGTCAACAACACCA CCACTATCC |
| Zebrafish *ofd1* | Forward | TAATACGACTCACTATAGGGATGTCTGCGAGTAAAG AGGAGAGT |
|  | Reverse | ATTAACCCTCACTAAAGGTCACCAGAAATCATCGTCGG |
